# Why Collect and Use Race/Ethnicity Data? A Qualitative Case Study on the Perspectives of Mental Health Providers and Patients During COVID-19

**DOI:** 10.3390/ijerph21111499

**Published:** 2024-11-12

**Authors:** Nancy Clark, Cindy Quan, Heba Elgharbawy, Anita David, Michael E. Li, Christopher Mah, Jill K. Murphy, Catherine L. Costigan, Soma Ganesan, Jaswant Guzder

**Affiliations:** 1 Department of Nursing, Faculty of Health, University of Victoria, Victoria, BC V8P 5C2, Canada; 2 British Columbia Operational Stress Injury Clinic, Vancouver Coastal Health, Vancouver, BC V5M 4T5, Canada; cindyquan@uvic.ca; 3 Department of Psychology, University of Victoria, Victoria, BC V8P 5C2, Canada; hebael@uvic.ca (H.E.); costigan@uvic.ca (C.L.C.); 4 BC Mental Health and Substance Use Services, Mental Health Commission of Canada, Vancouver, BC V6J 3M8, Canada; davidanita9824@yahoo.com; 5 Data & Analytics, Vancouver Coastal Health, Vancouver General Hospital, Vancouver, BC V5Z 1M9, Canada; michael.li@vch.ca (M.E.L.);; 6 Institute of Health Policy and Management Evaluation, University of Toronto, Toronto, ON M5S 1A1, Canada; 7 Interdisciplinary Health Program, St. Francis Xavier University, Antigonish, NS B2G 2W5, Canada; jkmurphy@stfx.ca; 8 Department of Psychiatry, Faculty of Medicine, UBC Vancouver Campus, Vancouver, BC V6T 2A1, Canada; soma.ganesan@soma-thera.com (S.G.); jaswant.guzder@mcgill.ca (J.G.); 9 Departments of Child Psychiatry & Social and Cultural Psychiatry, McGill University, Montreal, QC H3A 0G4, Canada

**Keywords:** mental health, COVID-19, ethnicity, race-based data, cultural safety, intersectionality, disaggregated data, racism, health equity, case study

## Abstract

Context: Calls to collect patients’ race/ethnicity (RE) data as a measure to promote equitable health care among vulnerable patient groups are increasing. The COVID-19 pandemic has highlighted how a public health crisis disproportionately affects racialized patient groups. However, less is known about the uptake of RE data collection in the context of mental health care services. Methodology: A qualitative case study used surveys with mental health patients (n = 47) and providers (n = 12), a retrospective chart review, and a focus group to explore healthcare providers’ and patients’ perspectives on collecting RE data in Canada. Results: The patient survey data and focus groups show that patients avoid providing identifying information due to perceived stigma and discrimination and a lack of trust. Providers did not feel comfortable asking patients about RE, leading to chart review data where RE information was not systematically collected. Conclusions: The uptake and implementation of RE data collection in mental health care contexts require increased training and support, systematic implementation, and further evaluation and measurement of how the collection of RE data will be used to mitigate systemic racism and improve mental health outcomes.

## 1. Introduction

Public health is built on the foundations of social justice and health equity [1]. However, the context of COVID-19 and the killing of George Floyd on 25 May 2020 have made it clear that social determinants of health such as racism remain a priority for making governments and institutions accountable for working toward health equity. Evidence suggests that COVID-19 has caused greater morbidity and mortality among racialized populations because of their perceived “race” and/or “ethnicity” [2,3,4,5,6,7]. Research highlights that people from ethnic minority communities may be more likely to experience higher levels of negative mental health factors such as distress, anxiety, stigma, and racism during the pandemic [8,9,10,11]. Despite the significant focus on the mortality and morbidity of racialized population groups, research about racialized patients living with pre-existing mental health conditions is an emerging area of investigation. Some evidence suggests that during COVID-19, Black and ethnic minority mental health patients did not have adequate access to mental health services and struggled with social connectedness and support, while also experiencing heightened anxiety, stigma, and racism associated with the pandemic [9,12].

In the context of the COVID-19 pandemic and its aftermath, many countries have adopted equity-based policies to systematically include the collection of race and ethnicity (RE) data to understand and eliminate racial and ethnic disparities in health care [13]. However, significant challenges to the full-scale adoption of these policies remain. Arguments supporting a lack of adoption of RE data include a lack of evaluation of improved health, willingness to address systemic racism, lack of community input, monitoring, and linking these data to population-level data to address existing health inequalities [14,15,16,17,18]. Moreover, distrust of how patient data are used may impede RE data collection, particularly in mental health settings, where discrimination already exists between patients and providers [19,20].

In Canada, the response to calls for a systemic uptake of RE data within health care settings has been slow, despite governmental legislation and public health authorities’ calls to adopt minimum standards for collecting race-based and Indigenous identity data in health systems, along with guidance on their use [20,21,22]. For example, a Canadian national survey conducted across 202 emergency departments (EDs) found that only 20.3% (41/202) systematically collected RE data, and 38.1% (77/202) reported that primary language data were systematically collected from all patients [23]. Similarly, a retrospective cross-sectional study using chart review data from a large tertiary academic hospital in Ontario, Canada, found that less than 50% of COVID-19 inpatients had a formal assessment of race or ethnicity and that the inclusion of race/ethnicity categories did not correspond to the government’s data standards [4].

Although race and ethnicity are distinct in meaning, they are often used together in research on race-based data to understand, monitor, and address social inequities in health [14,15,17,24]. Terms such as race, ethnicity, accessibility, and language (REAL) are used in studies to enable a meaningful use of disaggregated data collections across health information technologies [25,26,27,28]. In this study, both terms race and ethnicity (RE) are used to draw attention to health inequities caused by racism and the fact that ethnic minority groups are often racialized based on perceived differences.

The mental health of racialized population groups and the implementation of proposed standards for race-based data collection are not known. This study investigates mental health patients’ and providers’ perspectives on collecting RE data in a large urban hospital in western Canada during COVID-19. Understanding mental health providers’ and patients’ perspectives on why RE data collection is necessary can help explore the barriers to and facilitators of policy initiatives.

The research questions that guided this study are as follows: (1) What are patients’ and providers’ perspectives on why “race-based” data should be collected and used? (2) How are sociodemographic data being collected and used within a mental health inpatient clinical care setting?

## 2. Materials and Methods

### 2.1. Procedure

A qualitative, descriptive, case study methodology was applied to understand mental health patients’ and health providers’ perspectives on collecting RE data in the context of COVID-19. A case study methodology is defined as an inductive investigation bound by a system and unit of analysis or case, in which researchers collect multiple sources of data [29,30,31,32]. Case study research is typically described within specific parameters, e.g., place and time [31,32]. In this study, RE data collection within mental health practice settings provided the unit of analysis. The data sampling was purposive and included multiple sources of data collection such as survey data, chart reviews, and a focus group. All the data were collected from April 2022 to January 2023 from a large urban hospital in British Columbia (BC), Canada. The period of data collection defined the finite nature of the case study. For example, the retrospective chart review was collected during three phases of COVID-19 according to the pandemic phases defined by the implementation or relaxation of population-level mitigation measures in BC [33]. A critical theoretical orientation of social justice and equity informed the analysis and interpretation of the case study [34]. This included the concept of collecting disaggregated data, i.e., ethnic group, occupation, gender, and education, and assessing how they intersect to shape structural inequities [22]. Specifically, an intersectionality lens was used to understand how sociodemographic patient variables can be used within disaggregated data systems to redress social inequalities and improve health outcomes, particularly for patient groups who are impacted by multiple forms of discrimination [22,34,35,36]. This study included three steps: step (1) included collecting survey data; step (2) included focus group data; and step (3) included a chart review.

#### 2.1.1. Step 1: Survey Data

After receiving ethical approval, clinical directors for mental health units were contacted through a letter from the Coordinator of the Consumer Involvement and Initiative department asking if they would be willing to share this study through their hospital listserv. In addition, a recruitment poster about the study was developed and shared through psychiatric emergency departments, acute and short-term treatment units, and psychiatric outpatient treatment settings, e.g., Access and Assessment Centre (AAC) and Mental Health and Substance Use (MHSU) units. Staff (n = 12) and patient survey data (n = 47) were collected through an anonymous survey questionnaire. Mental health patients were also able to access the survey through a website called Spotlight on Mental Health, which is a not-for-profit organization for individuals with experience of accessing mental health services within the health authority. Participants (patients and providers) who completed the survey questionnaire also received information about how they could participate in a draw to win one of four gift cards valued at CAD 25. Both the patient and provider survey questions were designed to take approximately 5 min to complete and were developed by CQ. The survey questions included provider and patient ethnicities and were informed by best practice guidelines for self-reporting RE data [22,25,26,27,28]. Additional feedback for the survey questions were discussed with the research team until a consensus was reached. The provider and patient surveys can be found in [Supplementary Material A]. The survey data analysis was coded to identify key themes using a qualitative reflexive thematic analysis [37] coding template [Supplementary Material B].

#### 2.1.2. Step 2: Patient Focus Groups

Patients, providers, and family members who were interested in participating in a focus group could contact NC or CQ after completing the survey. Patients were also recruited through a recruitment poster advertised through Spotlight, an umbrella programme that amplifies the consumer/peer or service user’s voice within the hospital’s Community Mental Health and Substance Use Services. A patient partner consultant through Spotlight also shared the opportunity to participate in this study with prospective patients on a Consumer Involvement and Initiatives website and through word of mouth. Patients and family members of patients who were interested in participating, were over the age of 18, and had experience of accessing mental health care through the hospital were encouraged to participate. Interested patient participants who felt comfortable sharing their perspectives were later contacted by CQ and AD to confirm that they met the inclusion criteria. Six patients were recruited and participated in a focus group discussion. Patient participants for the focus group were given a CAD 25 honorarium. The focus groups were facilitated by a patient partner (AD) with experience in patient-oriented research and were hosted on Zoom. Interviews were recorded with permission for transcription. A set of focus group questions were co-developed by AD, CQ, and NC and used as a guide for the discussions [Supplementary Material C].

#### 2.1.3. Step 3: Chart Review Data

A cross-section of 200 charts were retrieved for patients admitted from January 2020 to May 2022. The charts were randomly retrieved by the hospital consultant for research advisory for data governance and data analytics through the hospital’s Data Release & Access Management Office. Electronic medical records were linked across the psychiatric emergency department, the Psychiatric Assessment Unit, and an inpatient unit which used two different electronic health record systems. A chart review data extraction tool was developed by CQ and NC to look for demographic information, e.g., age and sex, race, or ethnicity data (reported vs. unknown), and other demographic information, including marital status, housing status, employment status, sexual orientation, religion, and language [Supplementary Material D]. The reviewers (CQ and NC) initially conducted a pilot review of the data extraction tool by extracting data from 20 charts, which were subsequently discussed for determining the intercoder reliability (ICR). In this qualitative case study, ICR refers to consistency in capturing features of the data that were consistent with our data extraction tool [38]. This means that a consensus was reached between NC and CQ on whether the data extraction tool captured how RE were described in the patient charts. This approach is appropriate when categorizing data at a nominal level (e.g., presence or absence) in qualitative studies [38]. We included all patients who had been admitted to psychiatric emergency departments, acute and short-term treatment units, and psychiatric outpatient treatment settings, e.g., Access and Assessment Centre (AAC) and Mental Health and Substance Use (MHSU) units at the hospital, between the study’s start and end dates.

After pilot testing the data extraction tool, both NC and CQ conducted chart reviews and extraction separately at the patient data management unit within the hospital. The chart review data extraction was carried out over 2 days. A total of n = 84 clinical charts were retrieved, and a chart review table was developed for qualitative analysis [Supplementary Material E] and uploaded into an Excel file.

Transcriptions of the patients’ interactions with providers, including psychiatric consultations, progress notes, discharge summaries, and outpatient notes, were also reviewed to see if there was any additional information provided during the patient’s care, e.g., if the care or practice was informed of RE data. The sample size of the chart review data was determined based on its informational power, i.e., the adequacy of the number of charts was determined based on whether the sample was sufficiently large to answer our research question and aims [39]. Previous research on chart reviews have used sample sizes as low as n = 50 [19]. After a total of n = 84 charts, no new patterns of RE data capture were found.

### 2.2. Participants

The survey participants included both mental health patients/service users and multidisciplinary hospital staff (administrators, nurses, physicians, and social workers). Healthcare providers who did not work directly in mental health services or were not administrative staff were excluded. Only patients who received inpatient and/or community-based services for a mental health diagnosis and were over the age of 18 could participate. Patients who were experiencing acute illness were excluded. The participants also included those who provided support to a patient, e.g., family members (loosely defined, e.g., relatives, partners, and close friends). All survey participants were invited to be part of a focus group. Focus group conversations with six patients were conducted online over two Zoom meetings. A patient partner (AD) facilitated both discussions. The focus group participants consisted of mental health patients who received either inpatient or outpatient/community services from the hospital. The focus group participants’ self-identified ethnicities included Metis French Canadian, Chinese Canadian, Asian, Metis European, English Irish Canadian, and White Caucasian with German ancestry.

### 2.3. Materials

Three materials were used to collect and analyze the data and included two survey questionnaires, a chart review extraction tool, and a focus group interview guide. Both the mental health patient and provider surveys were developed by CQ and reviewed by the research team, who have expertise in developing questionnaires. The survey questions were developed using a broad range of literature on collecting disaggregated data from general populations. To understand patients’ and providers’ perspectives on why RE data should be collected, all participants were asked their views on why and how sociodemographic information should be collected, their perspectives as well as participant experiences on collecting RE data, and whether they thought it improved patient care. The patient survey questionnaire also asked about their experiences when they were asked to provide identifying information relating to their ethnicity to their healthcare providers. Based on the best practice for obtaining information about ethnicity, the survey participants were asked to self-identify their ethnicity through a write-in response question [35,36].

A chart review data extraction tool was co-developed by CQ and NC to look for demographic information, including ethnicity, marital status, housing status, employment status, sexual orientation, religion, and language. A chart review table was created [Supplementary Materials E] from an Excel file by HE to de-identify patient ethnicity data extracted from the health information records. Summary codes were developed to provide information about how ethnicity data were used in relation to culturally tailored care or support. This process was used to assist with analyzing the chart review and survey data.

A semi structured questionnaire [Supplementary Materials C] was used as a guide for the patient focus group. The questionnaire was co-developed by NC, CQ, and AD to gain a deeper perspective of patients’ experiences and included questions about COVID-19.

### 2.4. Analysis

Our findings were analyzed as individual units and then synthesized across all three data sets, i.e., survey, focus group, and chart review data, to identify common themes. A qualitative thematic analysis was used to develop analytic codes for the survey, focus group, and chart review data. A coding structure was used to develop patterns of meaning in each of the data sets. This inductive approach is consistent with qualitative thematic analysis [37], which helped identify both semantic codes, e.g., what people said, and latent codes, e.g., the deeper meaning and interpretation of the data. The semantic codes were clustered into themes or narratives that described the latent constructs. The consistency in data interpretation was evaluated based on the intercoder (ICR) reliability, sometimes called inter-rater reliability (IRR) [38]. In the qualitative analysis, the ICR was used to categorize the data at a nominal level, e.g., the presence or absence of RE data in chart reviews. In addition, the intracoder reliability was used to determine consistency in terms of how the same person codes data at multiple time points, which promotes researcher reflexivity [38]. For example, to determine the relevance of analytic themes across data sets, HE and NC held three consensus meetings. This process meets the minimum requirements for determining the clarity of conflicting interpretations [38].

Using an interpretive thematic analysis approach, we triangulated our findings across the three data sets and broader literature [37]. The survey data were analyzed by quantifying the perspectives of both providers and patients. As this report was exploratory with no predefined hypotheses, we report the descriptive statistics (proportions) of the survey data and chart review data alongside the qualitative data (qualitative survey responses and focus group discussion). The qualitative data from the open response survey questions were analyzed in relation to the descriptive statistics and coded by HE and NC to identify common themes and/or differences.

Data to identify patients for the chart review were extracted from the hospital’s Data and Analytics data warehouse holdings. The warehouse holdings were populated from source clinical systems across the services operated by the hospital in acute, primary, and community care. The initial patient cohort had an emergency department (ED) admission (between Mar 2020 to Mar 2022) with a Mental Health and/or Substance Use chief complaint or ED discharge diagnosis. The initial review of ethnicity field completion in the clinical systems showed a low data capture rate. At the time of analysis, ethnicity was not an available extractable field in the acute clinical system. Additionally, the best rate of data capture, which was still only a rate of 16.4%, was found in the community clinical system. A random sample of 200 patient records from the above cohort was then selected. In addition, patient records were only selected if they had at least one ethnicity field completed (even if “Unknown”) in a clinical system. From this cohort of 200 patients, 84 charts were manually reviewed. The chart review data collection was terminated at 84 charts, because we had determined informational power [37,39]. In other words, we were not finding any new patterns of how RE data were collected. To identify how demographic data were being collected and used within a mental health inpatient clinical care setting, a chart review data table was created to summarize the Excel files of demographic patient information obtained from the hospital’s electronic patient health records. This process allowed for the development of summary codes, with which we tried to capture any outcomes or interventions relating to ethnicity fields, e.g., culturally tailored support or other mental health interventions. The codes were developed and discussed by HE and NC to check for consistency of meaning and interpretations. The focus group transcripts were read individually by AD, NC, and CQ, and notes were made about the contents of the data. A consensus meeting was held with patients to validate and refine the themes from the focus group conversations.

## 3. Results

A total of n = 59 participants completed an anonymous survey. The demographic characteristics of the survey participants are presented in Table 1.

### 3.1. Mental Health Patient Survey Data

A total of n = 47 mental health patients completed the anonymous online survey. When asked what demographic information their health care providers asked for, 70% of respondents reported that they were asked about their age, 43% reported that they were asked about their gender identity, 41% reported that they were asked about their sex, 36% reported that they were asked about their preferred language, and 26% of respondents reported that they were asked about their ethnicity. About one in five (17%) patients reported that they were not asked to provide any of this information. Mental health patients were also asked their perspectives on the importance of collecting this information. Fifty-one percent believed it would improve their care, including by leading to culturally specific care, referral to specialists and relevant community supports, and receiving care that is appropriate for them. However, 38% of patients also expressed concerns about potentially negative consequences such as discrimination or racism. This was validated by some patients who responded to the following open-ended question: what is your opinion about sharing background information about yourself (e.g., ethnicity, sex, gender, age, etc., with healthcare providers?

*“I didn’t explicitly share my ethnicity and still experience labelling and stereotypes that are imposed on me”*—Patient 2.

*“I worry it could improve care for others, but if a health care worker has discriminatory beliefs it could also impair someone’s care”*—Patient 23.

When asked what information clients believe is important for their health care providers to know, a majority reported that age and preferred language are important (74.5% and 72%, respectively), followed by gender identity (66%), housing status (53%), and ethnicity (45%). Sex and religion were reported to be important by 31% and 28% of respondents, and additional information that clients felt was important included social support. When asked “What information do you think is important for health care providers to provide better mental health care for you?”, the qualitative responses included the following:

*“It depends on the reason for seeking care, the goals of care, and what’s important to the patient in terms of their identity and presenting concern”*—Patient 32.

*“Just because they know, doesn’t meant better care will be provided”*—Patient 40.

We found that 21 percent of patients felt more comfortable sharing their ethnicity with staff who are of the same ethnicity as them, while 13% felt more comfortable when the staff member is of a different ethnic identity. When asked “what makes you feel comfortable sharing your personal information with your health care provider”, the open-ended responses included the following:

*“I believe in an examined, conscious self-identification, respect, learned expertise as well as living experience and work in human rights, access and social justice as well as open mental health and substance use peer recovery without “cultural assumptions” and with priority of public health determinants, anti-colonialism and anti-racism”*—Patient 19.

Patients were also asked if they had a bad experience with sharing personal information with staff and what made them feel uncomfortable.

*“I was asked what my “Demographic” is, which confused me. I did not understand what that word meant at the time. During a mental health break. The receptionist continued to repeat the question to me even though I had said I did not understand the question. This happened until the point of me breaking out in tears”*—Patient 39.

*“When staff recognized one half of my ethnic/racial background but failed to document/mention the other half of my ethnic/racial background in reports. As a result, I felt like a huge part of the context of my mental health history was missing, and it felt awkward to have to try and fill in this missing information with future health care providers who were relying on these incomplete reports”*—Patient 12.

Overall, the survey responses from patients showed that they felt that collecting information about their ethnicity could improve their care but feared that the information could also lead to labelling and reinforce negative stereotypes. Patients believed they lacked information on why ethnicity data are being collected and felt more comfortable if the provider had lived experience and/or was of the same ethnicity.

### 3.2. Health Care Provider Survey Data

Twelve health care workers also completed the survey: seven clerical workers, four nurses, and one mental health clinician/social worker. The respondents reported that the demographic information they collected most from patients is sex and age (83% each), followed by language (67%) and gender identity (67%). Only 16.7% of providers reported collecting ethnicity data. Most providers felt that collecting demographic information helps increase the quality of patient care (83.3%), and 8.3% noted that understanding this information would inform their care and relate to the patient’s condition. When asked “What is your opinion on collecting demographic data from a patient?”, their qualitative survey responses included the following:

*“Understanding how this may be related to their illness and contribute to their care and treatment”*—Health care provider 11.

However, 25% felt that it collecting ethnicity was irrelevant to the quality of care. When asked what demographic information would be needed to help provide better care, most respondents believed that gender identity is of importance (92%), followed by language (83%) and age (75%). Ethnicity, religion, employment, and housing status were found to be of equal importance at 58.3%. When asked “What information do you think you need about patients to provide better mental health care?”, one provider mentioned

*“Social connections and social support”* (Health care provider 11).

Fifty-eight percent of providers believed that patients are reluctant to provide their demographic information due to privacy concerns. When asked “What barriers have you experienced when collecting sociodemographic data?”, the providers stated as follows:

*“…the client sometimes does not answer as this information is not a priority to them”*—Health care provider 10.

*“Admission template or admission form does not have all the areas identified in this form thus far”*—Health care provider 11.

Although most providers believed that collecting sociodemographic information could improve the quality of patient care (83.3%), privacy concerns, a lack of priority, a lack of training, and language barriers contributed to difficulties in collecting information about a patient’s ethnicity. In the chart review data, gender identity was perceived to be relevant for providing quality patient care, followed by language and age, which is consistent with the findings from the patient survey questionnaire. It also suggests that ethnicity data are less frequently asked about and that language may be a proxy for the provision of culturally tailored care, in addition to gender and age.

### 3.3. Chart Review Data

Our summary of the ethnicity categories in the chart review showed that 20.2% were identified as Caucasian without further identifiable ethnicities. This was followed by a range of other ethnicity categories. The remaining charts did not report an ethnicity or noted the ethnicity as “Unknown” [Table 2]. The retrospective chart review data showed that demographic data on ethnicity were not routinely collected, based on the available ethnicity categories in the patient admission notes (electronic medical record). It is important to note that, at the time of analysis, ethnicity was not an available category in the acute clinical system. Therefore, only ethnicity data from primary care and community care clinical systems were obtained.

Of the ethnicity fields that were available, 20.2% were identified as Caucasian, 14.3% as Aboriginal, 3.6% as First Nation, 2.4% as First Nation Status, 2.4% as Métis, and 1.2% as Métis non-status. The chart review data showed inconsistency in the terminology used for the ethnicity status of Indigenous groups in Canada. For example, Aboriginal was used as a descriptor in the chart progress notes and was not clearly linked to a specific Indigenous category such as First Nation, Métis, or Inuit as defined by the United Nations Declaration on the Rights of Indigenous Peoples (UNDRIP) [39]. Aggregating Indigenous people this way may not accurately reflect their self-identified ethnicity category. A summary of the ethnicity categories in the chart review data is presented in Table 3.

Of the n = 84 charts, 10.7% had missing data in the ethnicity fields. From the electronic health records that were reviewed, it was unclear if the ethnicity fields were completed with the patients or based on the provider’s perception only. Disaggregated data, which are other subcategories of identity such as language, culture, and family background, were found in 61.9% of the charts under the discharge and consultation notes.

The patient’s age and gender identity, marital status, and housing were the types of demographic information that were most often collected on admission, which corresponds with the survey questionnaire data findings. A patient’s housing status was noted in 98.8% of cases, and religion was found in 4.8% of cases. Marital status was included in 96.4% of cases, and some information regarding sexual orientation or current relationships was present in 22.6% of cases. Occupation, employment status, and socioeconomic status were rarely documented. For example, some chart notes only noted if the patient was financially supported by their parents or had a disability income.

Based on the chart review notes, referrals for community follow-up were made in 91.2% of cases, and approximately 17.9% of cases showed some form of cultural tailoring such as a translator or referral to a specialized community resource (e.g., counselling service for Indigenous patients). In terms of cultural tailoring based on ethnicity data, the chart reviews showed that Aboriginal (n = 3) and First Nations (n = 1) patients received some form of culturally tailored care, such as an Indigenous Patient Navigator or referral to culturally specific support.

Of the three charts with patients who were described as African, two were recommended for community mental health supports or psychosis-specific supports, and one did not have any referrals noted in their chart. For patients who were noted as Asian, Chinese, or Vietnamese, five charts described some form of culturally specific support, such as a translator for the patient or family or speaking with family members about the patient’s care. The remaining nine charts of Chinese patients described either general mental health supports, substance-/psychosis-specific supports, or no referrals being made. The chart of a Filipino patient showed that healthcare providers recommended counselling but did not report any culturally tailored care. One chart of a patient identifying as Punjabi showed that the health care providers spoke with their family and considered language-specific support, but the outcome of this was unclear. Another chart of a patient who identified as Southeast Asian only described previous support for psychosis-specific care but no other referrals. In general, many charts reported referrals to community mental health centres for follow-up.

In the context of COVID-19, most admissions were related to bizarre or paranoid behaviour (44%) followed by depression/suicidal/deliberate self-harm (25%). The patients’ mental health concerns as reported in the charts on admission are displayed in Table 4.

### 3.4. Focus Group Data

Focus group data were collected from six mental health clients who had either experienced inpatient or outpatient mental health services. Patients with experience of using services within the mental health system (inpatient or community) shared their perspectives on sharing their ethnicity and/or other parts of their identity within the mental health system and/or with their provider. In addition to recording patient perspectives on collecting ethnicity data, we also asked participants about their mental health in the context of COVID-19. To protect client confidentiality, names have been anonymized with pseudonyms. The focus group analysis was completed by NC, CQ, and AD. Data were coded through a descriptive thematic analysis process which included first reading the transcripts and summarizing key ideas in a Word document. Central themes that were captured were (1) client trust, confidentiality, and safety; (2) knowing why you are being asked; (3) stigma and stereotyping; and (4) promoting respect and culturally safe care.

#### 3.4.1. Client Trust, Confidentiality, and Safety

Patients were concerned about how the information would be used, given that they already felt stigmatized for having a diagnosis of a mental illness. As Barb explained,

*“The experience with having a huge stigma on my shoulder every time I would convey my diagnosis, and when I’m sick, I don’t trust anybody, and I have an incredible gap between me and the rest of the world when I’m sick. There’s not a lot of links for me to build a trust, relationship and by nature because I immigrated here on my own, and had to go through every decision, you know document and everything on my own. I’m extremely careful about how much I reveal”*—Patient.

The context of illness and stigma around having a mental health diagnosis is a barrier to sharing personal information with care providers. Similarly, Jenny stated the following:

*“And I think, especially in a mental health setting, it might be, you might come with a preconceived like mistrust of the authorities or someone who has … especially if it’s coming to the point where you might be taken into care against your will, or, like, you know, certified where you don’t have the option to leave if you want to. There’s a power imbalance and disclosing that or talking about that about your ethnicity or your cultural background might be really risky in those cases”*—Patient.

Patients were concerned about the inherent power imbalance in the medical system, particularly in contexts where they are receiving treatment against their will, e.g., being certified under the Mental Health Act. The implications of sharing their ethnicity could potentially lead to harm.

#### 3.4.2. Knowing Why You Are Being Asked

In most cases, patients discussed the importance of collecting sociodemographic information, including RE information. However, having a care provider explain the reason for collecting this could potentially improve trust and decrease barriers. As Annie explains,

*“I would say that knowing more about racial ethnicity can be important for mental health care. In that, I would feel comfortable, knowing that my care provider or my therapist understand sort of why I might do things. […] I mean my parents are very Chinese, but I’m not. […] I can feel comforted, knowing that my care provider sort of understands, you know, why I’m in this situation, […], it might be helpful”*—Patient.

Similarly, Billy explained the following:

*“Personally, the ethnicity is really important, but it can make me feel uncomfortable sometimes with how questions are asked… I think partly because I know that if we don’t record the information, nobody’s gonna know about you know all kinds of things, and the other is that also just on an individual level, it’s important for them to know who I am because it’s important to my own mental health issues”*—Patient.

Barb also stressed that

*“They need to be engaged to feel comfortable. So again, …, everybody is so unique and different, and you cannot just have a cookie cutter, “These are the questions you should ask everybody” because not everybody, react the same way and for me, if you ask me something that to me is irrelevant, it makes me suspicious”*—Patient.

Providing information about how patient data will be used was important for the focus group participants. Importantly, knowing when information should be asked and how it is shared was stressed as important for upholding patient rights.

#### 3.4.3. Stigma and Stereotyping

Fear was a major barrier for patients in terms of disclosing personal information about their ethnicity. Patients described that information could lead to stigma and stereotyping. Barb explained that

*“Treating the biases of the people that are healthcare providers, and that are doctors, of them understanding to treat people with respect and dignity, no matter where the mental illness is and not a matter of what ethnicity…”. “I almost feel that by collecting so much data on race, and putting people into boxes, it actually creates more racism almost by you know the underlying compartmentalizing people and I’m just not only you know boxed in, but you also know my ethnic background”*—Patient.

Similarly, Jenny added the following:

*“Yeah, think it’s a natural human instinct or preference to put labels on people and it’s just another way, it’s the same with diagnosis. Oh, this person’s bipolar or this person has borderline personality. It should ideally be informative, but not definitive. And so, you look at a label and you say, “Oh, this person is white, this person’s Indian, this person’s bipolar, this person… and you already have formed this assumption and everything you see is colored by those assumptions. So, then you have the cognitive bias and then so it’s like, in some way you almost don’t want to have any kind of labels”*—Patient.

Patients discussed that having a diagnosis already labelled them and perpetuated stigma and feared that disclosing their ethnicity could perpetuate stereotyping and bias amongst providers.

#### 3.4.4. Promoting Respect and Culturally Safe Care

The patient focus group participants stressed that overall, their human rights entailed a need for respect and cultural safety. Potential harm could be caused when only one aspect of their identity was collected. Barb stated that

*“The person that was there was the head nurse and another staff. And when I shared with them, like the trauma and stuff that is experienced for people that actually are going through that, and what should be done different and how to treat people differently, with respect and dignity. Asking about race/ethnicity will not automatically promote culturally safe relevant or equitable care, it may also be harming. Judgements can be made when only one aspect of sociodemographic information is collected”***—**Patient.

Similarly, Connie added that

*“It’s because of mental health. And as a result, I think being asked myself, and I like the term that Jess used “white passing”. Some people just, you know, I mean I will be asked by patients on many occasions, like, “do you have native in you or are you Indigenous?” … If you want to ask people about their background and their ethnicity, I think it should be a language that should be non-offensive, safe and kind. And what do I really need to know? I mean, is this person being effective as a result?”*—Patient.

The patients’ perspectives suggest that culturally unsafe practices occur when people are not treated with dignity and respect. In addition, simply asking does not mean that patients would receive culturally safe, equitable, or quality care, as Billy explained:

*“That’s […] a problem that needs to be addressed, no matter who they are, or what their background is. But when we look at things, at the at kind of a larger level, you know, we might notice things that, like maybe people of a certain ethnic group are exposed to greater rates of certain forms of violence or mistreatment in mental health care or denied certain access. And I think, and especially when we look at the history of mental health care and the way that race has played, and how groups of people are treated. You know, like there’s certain legacies that I think like and that’s where I think it goes beyond the you know, “Oh, we’re just doing it to make us look good”*—Patient.

Despite the barriers to collecting RE data perceived by the patients, they felt that there needed to be a follow-up beyond simply asking about their identity and its relevance to their care.

### 3.5. Overall Findings

A case study inquiry investigates a given case to address the “how” or “why” questions concerning the phenomenon of interest [30]. The multiple data collection methods helped us understand patients’ and providers’ perspectives on why “race-based” data should be collected and used and how demographic data are currently collected within a mental health inpatient setting. The triangulation of all three data sets showed consistent findings. First, the survey data on providers’ perspectives showed that ethnicity was not prioritized, even though it was considered an important part of patient care. Barriers to collecting ethnicity data included privacy concerns, a lack of priority, a lack of training, and language barriers. The patient focus group and survey data stressed concerns about stigma, stereotyping, and the potential for racism and discrimination. A lack of trust in care providers and historical harms experienced by mental health patients underscored their fear of disclosing their ethnicity. When, how, and for what purposes patient information was processed was perceived by patients as important to their human rights and the provision of culturally safe and equitable care.

Ethnicity categories were not consistently completed in the patient charts. The chart review data did not show any impacts of COVID-19 on mental health patients, i.e., there were no progress notes indicating that patients were having increased mental health problems. In the COVID-19 context, there may have been other factors that prevented the full-scale adoption or implementation of RE data collection, such as structural barriers and public health efforts to prevent the spread of COVID-19 and promotion of immunization uptake.

## 4. Discussion

In 2020, the Canadian Institute for Health Information (CIHI) produced pan-Canadian standards for collecting race-based and Indigenous identity data in health systems as a response to measuring health inequalities (also referred to as health disparities) experienced by racialized population groups during COVID-19. The findings from this study suggest that there is currently a lack of infrastructure to support RE data collection within and across acute and community mental health care settings. Our findings showed a lack of provider knowledge and privacy concerns, which is consistent with other research that also demonstrates other barriers to race-based data collection, such as a lack of community governance and a lack of monitoring and evaluation of health inequities and strategies to mitigate racism [14,15,21,22]. This study supports the conclusion that implementation strategies for collecting RE data must include the meaningful involvement of patients, communities, and multiple stakeholders to promote transparency and equitable power structures. The structural conditions must include infrastructure for leadership, resources, accountability, and evaluation strategies for reducing health disparities amongst racialized population groups [14,15,16].

The perspectives of patients and health care providers show that collecting RE data is important. The patient survey data were consistent with the care provider reports, showing that 83.3% believed that collecting demographic information would increase the quality of patient care. Similarly, the patient survey data also showed that 51% believed that collecting demographic information would improve their care, potentially leading to culturally specific care, referral to specialists, and relevant community support. However, fear and a lack of knowledge and trust were barriers to collecting ethnicity data. However, collecting disaggregated patient data, including RE data, offers a way to monitor and measure health inequities and disparities [14,15,21,22] and assess whether mental health patients experience multiple forms of discrimination based on intersecting dimensions of racism such as their gender, disability, mental health status, and ethnicity.

In this study, COVID-19 impacts did not emerge as significantly contributing to the patients’ or providers’ perspectives. This was a surprising finding given the disproportionate impacts of COVID-19 on racialized population groups [9,12]. However, this finding could be related to the fact that we did not have specific questions about COVID-19 and its impacts on racialized mental health patients. It may also be related to the fact that racialized mental health patients experienced more isolation during COVID-19 [8,9,10]. The patient and focus group data suggest that patients with pre-existing mental health conditions experience multiple dimensions of discrimination due to stigma around mental illness, which may surpass the impacts of COVID-19. The patient survey data showed that 38% believed that sharing their ethnicity could potentially lead to negative consequences such as discrimination or racism. This is consistent with studies reporting that people with mental health problems belong to more than one marginalized or stigmatized category, including racial–ethnic minority groups [10,40,41]. Despite these legitimate concerns, patients also stand to gain the potential improvement of their health care experience, by sharing their ethnicity, patients can also contribute to mitigating systemic racism and receive appropriate health care interventions [14,15,16]. The implications of this study suggest that mental health patients who also experience racism may also accept or reject COVID-19 vaccinations based on their relationship, trust, and experience with the health care system. Ethical issues relating to vaccine administration and decision making are related to free will and embedded in power relations [42]. Power differentials are particularly relevant for mental health patients who are certified for treatment [43,44]. Patient recommendations for collecting RE data include that providers share information with patients about why they’re asked and what will be done with the information and consider the timing of questions so that they are not asked in an acute phase of illness. The New Zealand Government Ministry of Health, for example, has developed protocols for asking standard ethnicity questions which include access and disability factors, such as providing questions in a sizable graphic with a large font size that is obtained during a health encounter or interview [45]. In addition, providing different processes for collecting ethnicity such as self-completion (paper or electronic form/questionnaire), verbal response, assisted response, and proxy response could improve patient responses. In each process, patients are provided access to an interpreter if necessary, and questions are followed up on if not answered [45]. These recommendations are consistent with recommendations to mitigate potential harm related to discrimination and data privacy [19,46].

Policy guidelines recommend increased transparency and participatory approaches to disaggregated data collection [14,21,22]. The United Nations Human Rights Office of the High Commissioner (OHCHR) (2018) has adopted a human rights-based approach toward implementing policy guidelines on disaggregated data collection [46]. The guiding sets of principles stem from the 2030 Agenda for Sustainable Development (2030 Agenda) and its Sustainable Developments Goals (SDGs) to leave no one behind. Principles include capacity building and increasing literacy and understanding of the purpose and process of disaggregated data collection within community groups and those who are directly affected by social inequalities. These principles underscore many of the policy initiatives and recommended guidelines amongst countries with colonizing histories such as Canada, the US, the UK, and New Zealand. The Agenda for Sustainable Development 2030 principles could promote better uptake of disaggregated data collection for health care institutions where Indigenous and racialized communities living with mental health conditions still fear that their data may be improperly used [19,24,40].

The chart review data showed that ethnicity fields were not systematically completed, and even when they were, there was a lack of information about the quality of care that patients received, i.e., culturally tailored care, resources, or support. This makes it difficult to measure and evaluate the relationship between ethnicity and health outcomes for patients [15,16,17,47,48]. 

In this study, 58% of providers believed that patients were reluctant to provide information due to privacy concerns. Only 16.7% of providers reported collecting ethnicity data. Providers also felt uncomfortable asking questions about a patient’s identity because of lack of training, language barriers, and a belief that ethnicity data are not required upon admission. The current best practice guidelines state that collecting disaggregated data, particularly for racialized patient groups, in a safe, respectful process can mitigate potential harms [20,44]. Health care provider education and training must include guidance on how to facilitate culturally safe RE data collection. This can include developing scripts for providers, clear communication with patients as to why RE data are collected and providing culturally appropriate services for patients to help manage potential harms [20]. Providers must be able to explain the benefits and risks of RE data collection and give patients the choice to provide or not provide self-identifying information [20]. Patient-friendly data collection procedures must treat people with autonomy and respect by allowing patients to opt out of questions and using categories that align with people’s identities, and which include options for a person to change their categorization [14]. Our study showed that 21% of patients felt comfortable sharing their ethnicity with providers from the same ethnic background. However, it could also be the case that ethnic matching may re-stigmatize mental health patients within their own ethnic groups. Other research suggests that there is a need for representation from Black, Indigenous, and other racialized medical professions to address racism and colonialism [49,50,51].

Research in the US showed that a higher level of support for collecting language information compared with collecting race and ethnicity data may result from patients more readily seeing how collecting language information could improve access to interpreters and increase the safety and quality of health care [52]. The providers’ perspectives in this study showed that 92% believed that collecting information about gender identity was important, followed by language (83%) and age (75%). Similarly, the patient survey data showed that age and preferred language are important (74.5% and 72%, respectively), followed by gender identity (66%), housing status (53%), and ethnicity (45%). Like public health policies on race-based data collection, the implementation of medical language interpreters remains a fundamental human right but has not been widely adopted across the provider, organization, or system levels [52]. However, when race-based data policies combine race, ethnicity, and language demographics, they may be more likely to address health disparities amongst patients [25,26].

Patients’ human rights and respect for patients are especially significant given the historical harms that mental health patients have experienced across health care institutions. The patient focus group data suggest that mental health patients can feel stereotyped when they are lumped into one ethnic identity without consideration of other demographic factors shaping their mental health and access to care. Lumping identities together can become exclusionary and conceal crucial differences within groups with respect to equitable mental health outcomes. It is therefore important to capture disaggregated data to reveal inequalities and relationships between each demographic category [16,22].

Many patients belong to multiple, overlapping communities and ethnicities, so it is important to capture granular-level data to capture the subcategories of demographic patient information [16,53]. Increasingly, the lens of intersectionality is used to understand demographic patient data collection and its relationship with health inequalities [21,22,54]. Natural Language Processing (NLP) tools offer free text options for patients to self-report and can be used to address multiple identity categories [55]. Self-reported ethnicity data are the “gold standard” and therefore, more valid than data on ethnicity collected by health care providers [25,26].

Our findings showed that most demographic data are collected at intake or admission by clerical workers (58.3%), nurses (33.3%), and mental health clinicians/social workers (8.3%). It is therefore important that race-based data policies include training and infrastructure resources to achieve the gold standards for RE data collection across all levels within institutional organizations, including health information technology systems such as electronic health records, to promote continuity and quality of care [26,55].

### 4.1. Limitations

The sample of our survey data was small and lacked provider heterogeneity. Obtaining diverse interdisciplinary perspectives may have provided further insights into how RE data might address the quality of care and patient health outcomes. Although the survey data were descriptive and not intended for generalizability, we did not include specific questions about the impacts of COVID-19 and mental health. This may have provided further insights into the context of COVID-19 and the ethnic background of patients and providers. Furthermore, collecting descriptive RE data does not provide an understanding of how solutions aimed at health equity can be measured across different racial identities. In addition, the case study method may have limited a more in-depth understanding of the barriers, as well as facilitators, for the implementation of RE data collection.

Although family members are an important part of patient care, especially when the patient speaks limited English, we were only able to recruit clients with direct experience and no family members. Providers also had options to participate in focus group discussions; however, only survey questionnaires were obtained. More focus groups with mental health patients may also have increased the voice and validity of our findings. However, this study was conducted during COVID-19, which may also have created barriers to recruitment. This study does provide some insight into mental health patients’ and providers’ perspectives on collecting RE data in the context of COVID-19, which can be used for future research on RE data collection across public health systems and institutions advocating for race-based data collection.

### 4.2. Implications and Future Directions

Mental health patients’ and providers‘ perspectives on why RE data should be collected suggest that collecting RE data is an important part of care, but only if it is considered in relation to other disaggregated data such as gender, disability, sexuality, age, and language. Importantly, disaggregated data that include RE alongside other demographic data are important tools for actions aimed at health equity [15,16]. Having processes which measure mental health equity and quality care rests on having reliable data. At the time of this research study, the wide adoption of RE data collection within the studied health care institution was not integrated across emergency departments and mental health units, as well as primary care and community care settings, with which the chart review data capture was linked, thus impacting the gaps and inconsistencies found in the retrospective chart review. Despite policies aimed to provide guidance on race-based data collection, there is limited infrastructure within health care settings to implement race-based policies. Future research could focus on the institutional capacity to adopt and implement race-based data policies. The implementation of scientific frameworks can be used to identify barriers, facilitators, and factors that promote institutional capacity for race-based data collection to promote health equity [56,57].

Mental health patients experience multiple intersecting discrimination processes based on stigma and negative experiences with the health care system. There is a need for increased provider and patient education and training on how identity questions are asked, as well as explicit information about how this information is shared and for what purpose. Training and education must include patient voices alongside multiple interdisciplinary perspectives, including health informatics and administrative staff’s perspectives. Building from this study, there is a need to examine the impact of patient–provider racial concordance and factors that support trust during mental health encounters. For patients with mental health conditions, asking questions during the acute stages of ill health was perceived as disrespectful and exacerbating distress. Mental health patients are already stigmatized and are distrustful of the mental health system as a result, meaning that knowing how and when to ask is important to mitigate fear and potential harm. Future research should expand outside of mental health care settings to evaluate how other departments are implementing race-based information and policies, as well as the factors that support patient trust during clinical care encounters.

## 5. Conclusions

This qualitative case study offers insights into why RE data should be collected from the perspective of mental health patients and providers. Our findings showed that both patients and providers believed that collecting RE data could promote better-quality care. However, patients believed that collecting information about their ethnicity could also cause harm, reinforcing stereotyping and discrimination. Increased healthcare provider transparency about why, when, and how questions are asked can facilitate trust and better compliance with standards for race-based data collection. The providers did not feel comfortable asking patients about their ethnicity. Measuring and using patient RE data must lead to mitigating systemic racism and improving health disparities for mental health patients. The effects of COVID-19 have exposed the intersections of racism and mental health as significant public health challenges. Increased training and education alongside measuring implementation outcomes will advance our understanding of the implementation of RE data standards and processes.

## Figures and Tables

**Table 1:** Survey respondent demographics.

	Patients		Providers	
Demographic Variable	N	%	N	%
Sex				
Female	35	74.5%	9	75%
Male	10	21.3%	2	16.7%
Blank	2	4.2%	1	8.3%
Gender (cis)				
Women	33	73.3%	9	81.8
Men	8	18.2%	2	18.2%
Gender Minority/	4	9.1%	N A	N A
Unspecified				
Sexual Orientation				
Heterosexual	23	48.9%	11	90.9%
Gay	Not available	Not available	1	9.1%
Lesbian	2	4.3%	NA	NA
Bisexual	5	10.6%	NA	NA
Pansexual	3	6.4%	NA	NA
Queer	5	4.3%	NA	NA
Prefer not to say	8	17%	NA	NA
Two-spirit	1	2.2%	NA	NA
Ethnicity				
African	1	2.3%	NA	NA
Caucasian	21	47.7%	3	25.1%
East Asian	8	18.2%	1	8.3%
South Asian	1	2.3%	4	33.3%
Southeast Asian	1	2.3%	4	33.3%
Indigenous	3	6.8%	NA	NA
Multiple	9	20.5%	NA	NA
Ethnicities				
Age				
Mean (SD)	43.6 (14.8)		38.3 (14.1)	
Age Range	16–81		23–70	

NA = Not available.

**Table 2:** Summary of ethnicity categories in chart review.

Ethnicity Category	N (84 Total)	Percentage
Aboriginal	12	14.3%
African	3	3.6%
Asian	1	1.2%
Blank	9	10.7%
Canadian	10	11.9%
Caucasian	17	20.2%
Chinese	10	11.9%
Filipino	1	1.2%
First Nations	3	3.6%
First Nations Status	2	2.4%
French	1	1.2%
German	1	1.2%
Metis	2	2.4%
Métis non-status	1	1.2%
Other	1	1.2%
Punjabi	1	1.2%
Southeast Asian	1	1.2%
Unknown	7	8.3%
Vietnamese	1	1.2%

**Table 3:** Available race/ethnicity categories in medical patient records.

Primary Care Clinical System	Community Care Clinical System
Aboriginal African Arab Asian (not further defined) Black Caucasian Chinese Declined European Filipino First Nations Indian Indigenous Inuit Japanese Korean Latin American Latin American/Hispanic Metis Middle Eastern Not Stated Other Other Asian South Asian Southeast Asian Unknown West Asian	Aboriginal African Arab Armenian Asian Black Canadian Caribbean Caucasian Central European Chinese Eastern European Filipino First Nations First Nations non-status First Nations Status French German Greek Hispanic Indigenous Inuit Inuit Status Inuit non-status Iranian Irish Italian Jamaican Japanese Korean Latin Latin American Lebanese Metis Metis non-status Metis Status Other Portuguese Punjabi Russian Scandinavian Somali South American South Asian Southeast Asian Southern European Unknown Vietnamese

**Table 4:** Summary of main presenting mental health concerns upon admission.

Main Category of Admission	N (84 Total)	Percentage
Mental Health		
Bizarre/paranoid behaviour	37	44.05
Depression/suicidal/deliberate self-harm	21	25.00
Hallucinations/delusions	5	5.95
Anxiety/situational crisis	4	4.76
Violent behaviour	3	3.57
Homicidal behaviour	1	1.19
Altered level of consciousness	1	1.19
Substance Use		
OD ingestion	6	7.14
Substance withdrawal	2	2.38
Other		
Laceration/puncture	1	1.19
Medication request	1	1.19
Minor complaints NOS	1	1.19
Sore throat	1	1.19

## Data Availability

Data are unavailable due to privacy and ethical restrictions. The data sets presented in this article were adapted into tables taken from hospital administration data, and original hospital administration data are not readily available because they include patient health care numbers. Requests to access the data sets should be directed to the data analytics and ethics department of the health authority, where the clinical data were retrieved from, and the corresponding authors C.M. and M.L.

## Supplementary Materials

The following supporting information can be downloaded at: https://www.mdpi.com/article/10.3390/ijerph21111499/s1, Supplementary Material A–E.
