# Supplementary material for: Why Collect and Use Race/Ethnicity Data? A Qualitative Case Study on the Perspectives of Mental Health Providers and Patients During COVID-19
Source: Int J Environ Res Public Health. 2024 Nov 12;21(11):1499. doi: 10.3390/ijerph21111499 (PMC11593584; doi:10.3390/ijerph21111499)
Supplement: Supplementary file 1 [file ijerph-21-01499-s001.zip › ijerph-3194440-supplementary.pdf]

Supplement A

A.1 Survey Questions-Clients

1. I am responding based on
    a. My personal experience receiving care at the [name of hospital]
    b. A family member's experience receiving care at the [name of hospital]

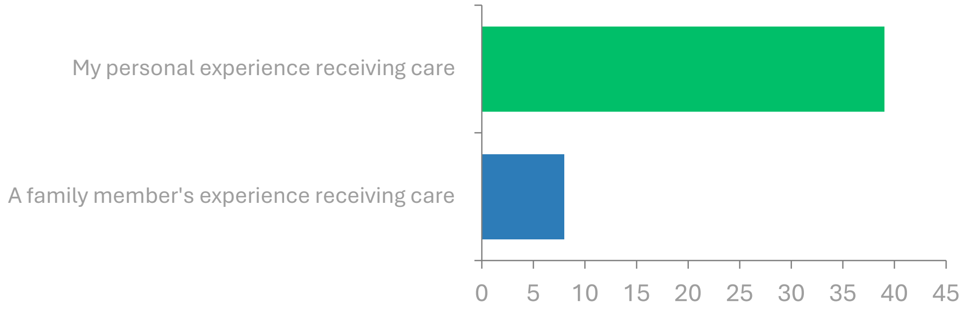

2. Thinking about times when you've received care at [name of hospital], where you asked to share information about the following (e.g., at intake or on a form)? Choose all that apply.
    a. Ethnicity
    b. Age
    c. Employment
    d. Preferred Language
    e. Sex
    f. Gender
    g. Housing Status
    h. Religion
    i. I was not asked to provide this information
    j. Other (please specify)

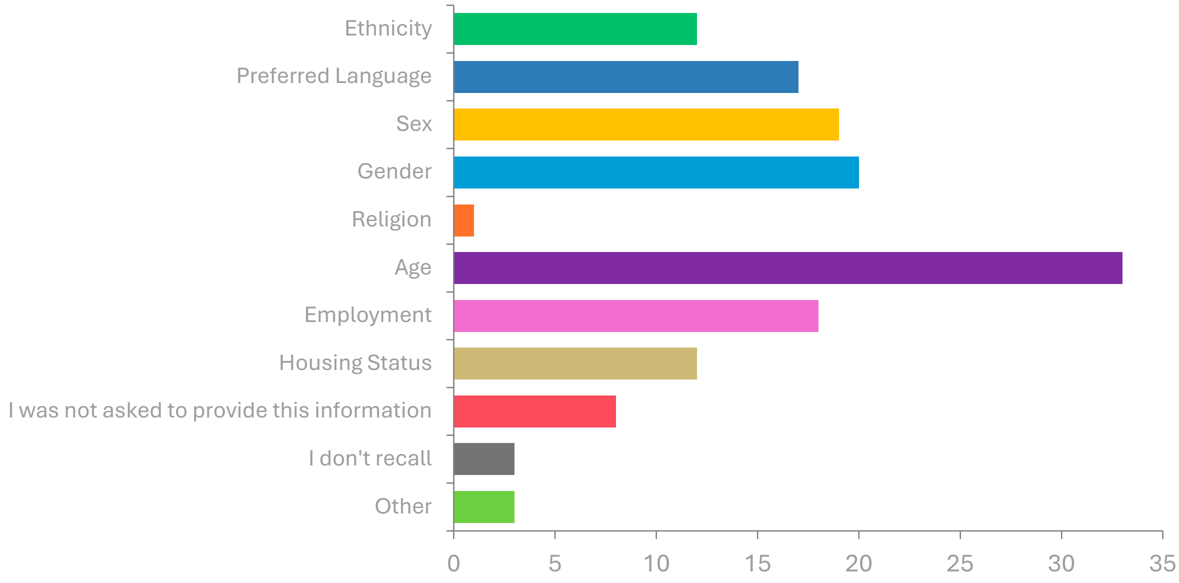

3. What is your opinion about sharing background information about yourself (e.g. ethnicity, sex, gender, age, etc.) with healthcare providers? Choose all that apply.
    a. It can improve the quality of care I receive
    b. It does not matter for the quality of care I receive
    c. I worry that I might experience negative consequences, such as discrimination of racism, if I share this information
    d. I worry about my privacy when sharing this information
    e. Other (please specify)

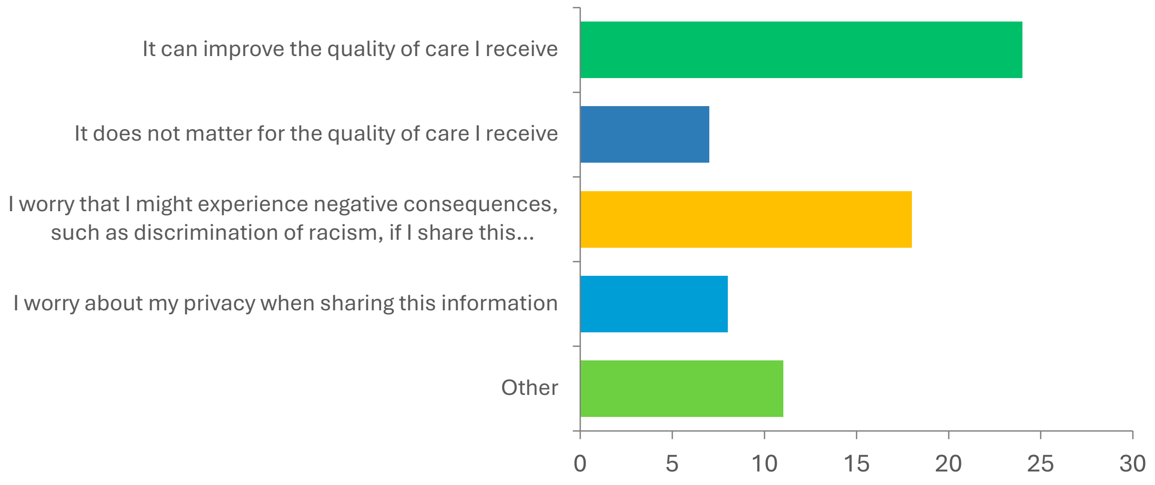

4. What information do you think is important for health care providers to have to provide better mental health care for you? Choose all that apply.
    a. Ethnicity
    b. Age
    c. Employment
    d. Preferred Language
    e. Sex
    f. Gender identity
    g. Housing Status
    h. Religion
    i. Other (please specify)

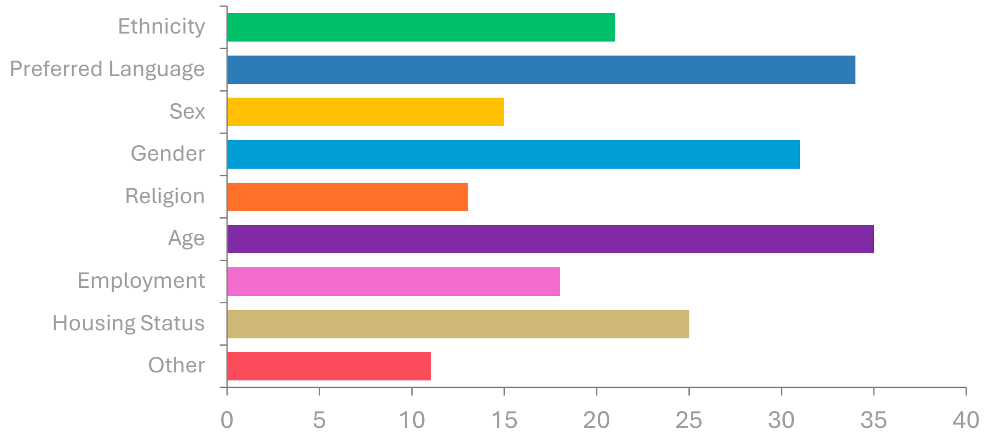

5. How does knowing about your background improve your mental health care, if at all? Choose all that apply.
    a. Health care providers have a better understanding of me
    b. It can lead to referral to specialists and community support (e.g., language interpreters or cultural bridges) that better fit my needs
    c. It can help me receive care that is appropriate for my culture, gender or background
    d. I don't believe that is does improve my mental health care
    e. Other (please specify)

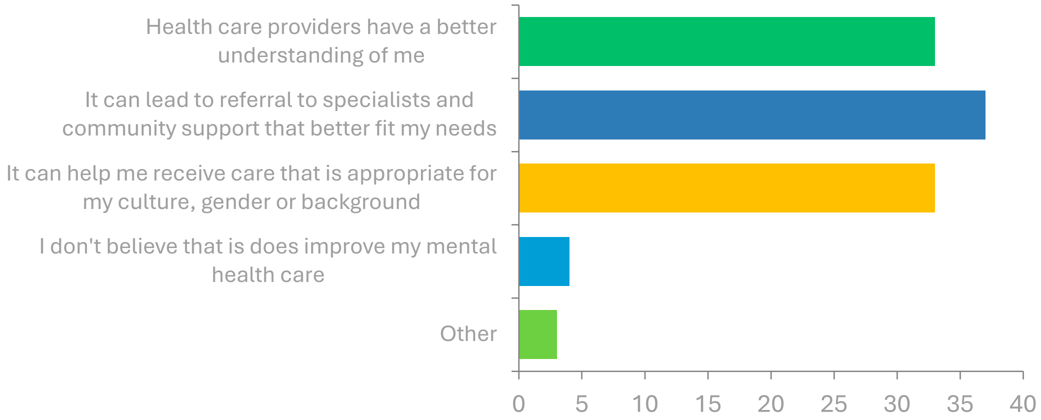

6. If you have provided background information about yourself to a health care provider, what was your experience of sharing the information? Choose all that apply.
    a. Someone explained why they collected the information
    b. I was concerned about why they were asking
    c. No one explained why they collected the information
    d. I was glad they were asking
    e. I barely noticed; it felt routine
    f. I have not been asked socio-demographic questions
    g. Other (please specify)

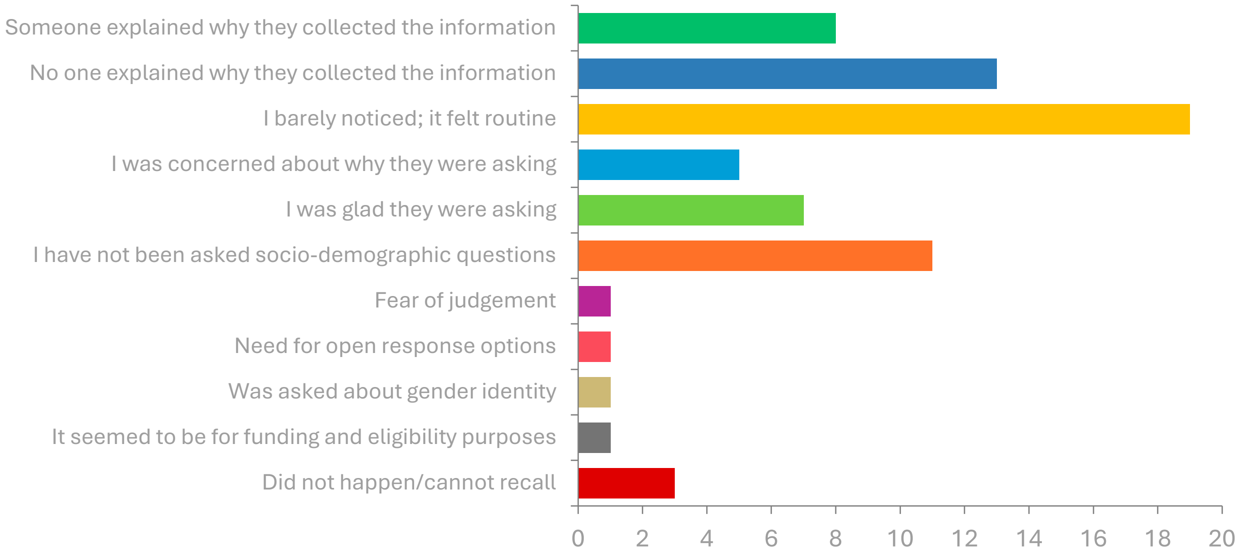

7. What makes you feel comfortable sharing your personal information with your healthcare provider? Choose all that apply.
    a. The staff and I share a similar ethnicity
    b. The staff and I have different ethnicities
    c. The staff and I speak the same language
    d. The staff and I speak a different language
    e. The staff explains why they are asking about my background
    f. I have had a good experience in the past when sharing my personal information
    g. None of the above matters for feeling comfortable sharing my personal information
    h. Other (please specify)

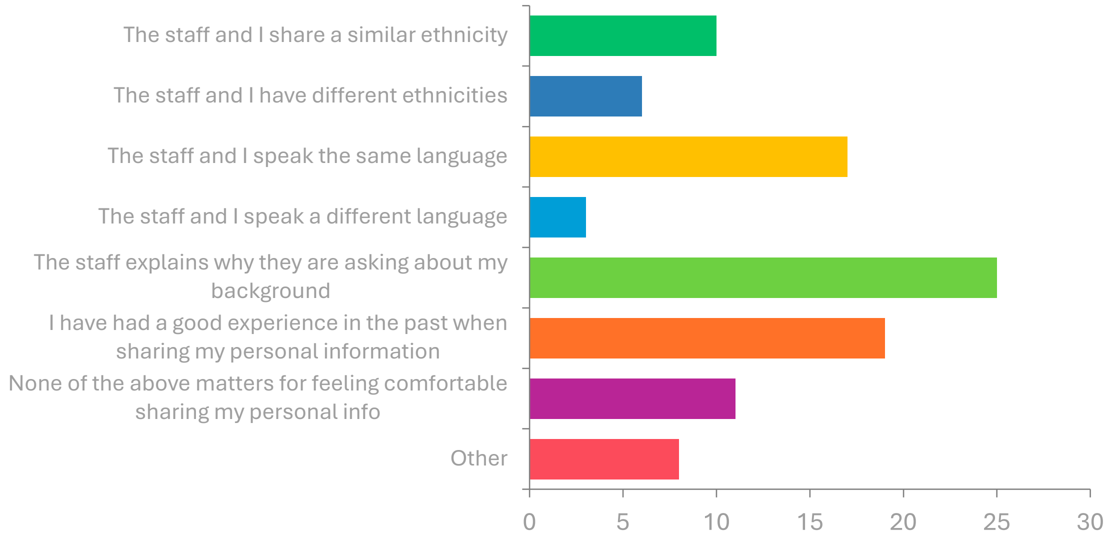

8. If you had a bad experience sharing your personal information before with a staff, what made you feel uncomfortable?

9. What mental health services did you or do you receive? Choose all that apply.
    a. Outpatient services in a clinic for mental health
    b. Peer support for mental health
    c. Inpatient in the hospital for mental health
    d. treatment for addiction / substance use
    e. Community mental health services (e.g., Assertive Community Treatment (ACT))
    f. Other (please specify)

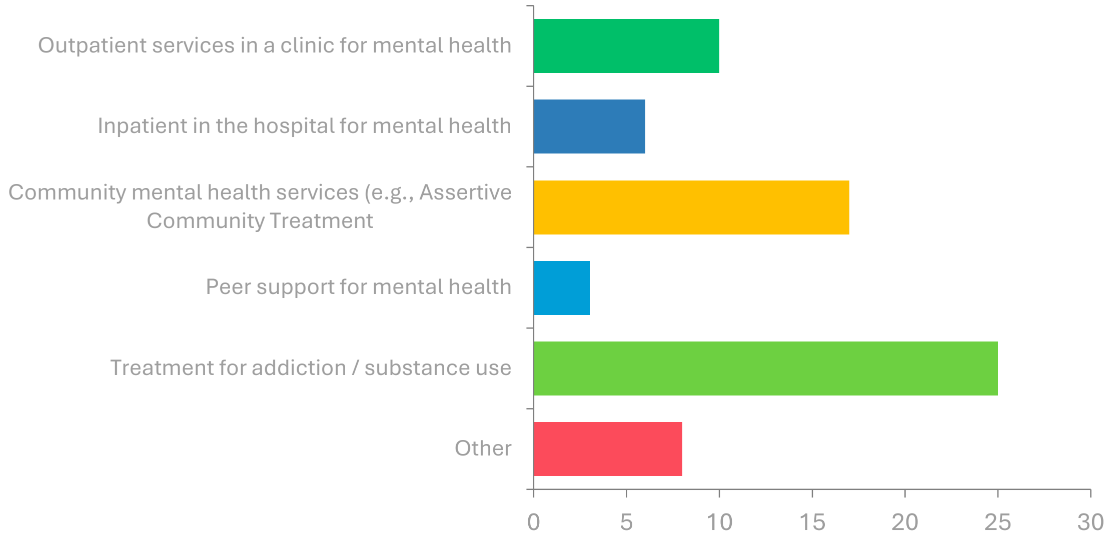

10. What are your ancestor's ethnic or cultural origins? Specify as many origins as applicable. Ancestors may have Indigenous origins (e.g., Cree, Métis, Inuit), or origins that refer to different countries (e.g., Scottish, Chinese, Jamaican, Moroccan) or other origins that may not refer to different countries (e.g., Jewish, Acadian, Punjabi). For additional examples, refer to this list of ethnic or cultural origins .

11. What is your current age (in years)?

12. What is your sex?
    a. Male
    b. Female

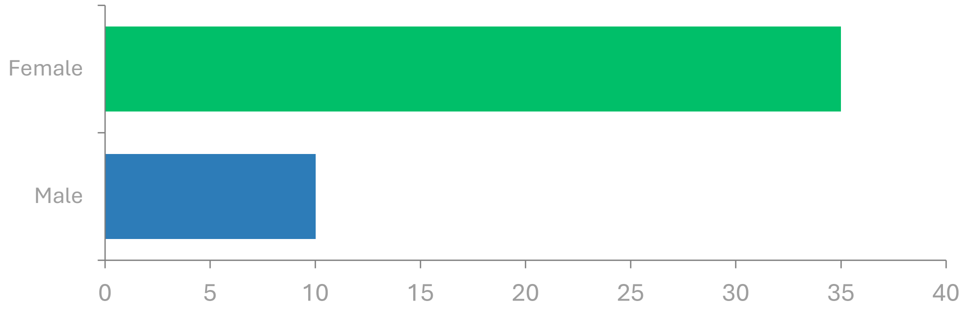

13. Is the gender you identify with the same as your sex registered at birth?
    a. Yes
    b. No
    c. Prefer not to answer
    d. Other: self-describe your gender identity

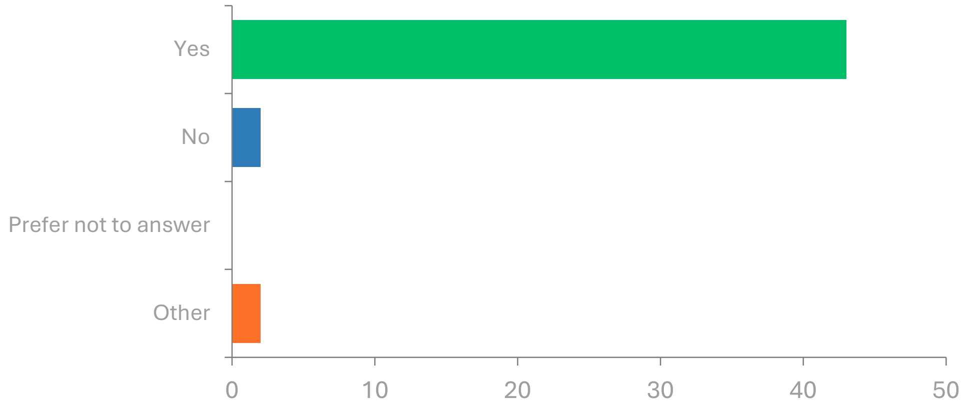

14. What is your sexual orientation?
    a. Asexual
    b. Bisexual
    c. Gay
    d. Heterosexual or straight
    e. Lesbian
    f. Pansexual
    g. Queer
    h. Prefer not to answer
    i. None of the above, please specify

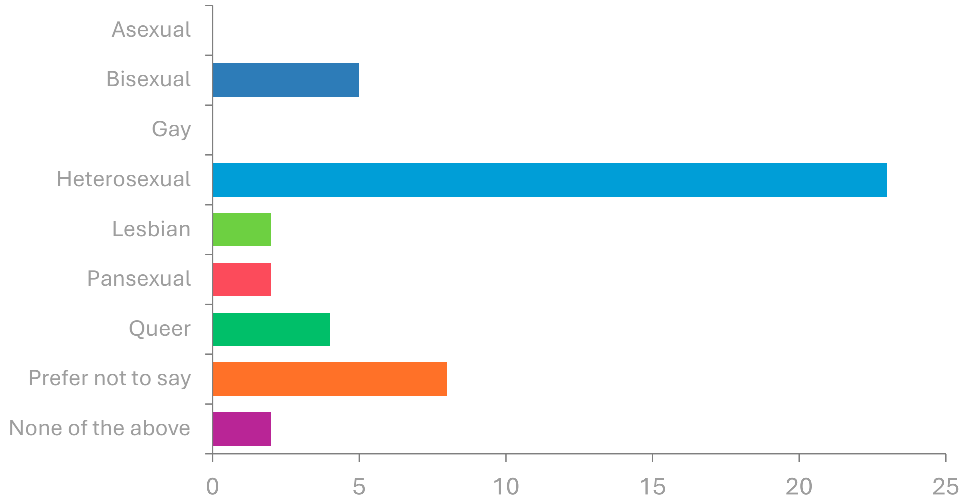

15. Have you ever experienced discrimination (i.e., denied equal treatment) based on any of the following characteristics or membership? Select all that apply.
    a. Ethnicity
    b. Age
    c. Employment Status
    d. Language
    e. Sex
    f. Gender
    g. Housing Status
    h. Sexual Orientation

i. Religion
j. Other (please specify)

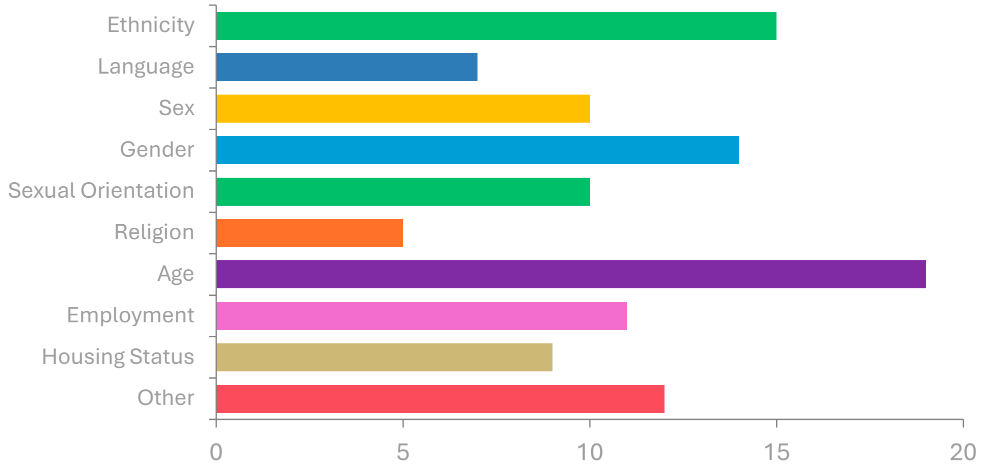

A.2 Survey Questions-Staff

1. In your role, what, if any, patient sociodemographic data do you collect? Select all that apply.
    a. Ethnicity
    b. Language
    c. Sex
    d. Gender Identity
    e. Religion
    f. Age
    g. Employment
    h. Housing Status
    i. I do not collect this type of information as part of my role
    j. Other (please specify)

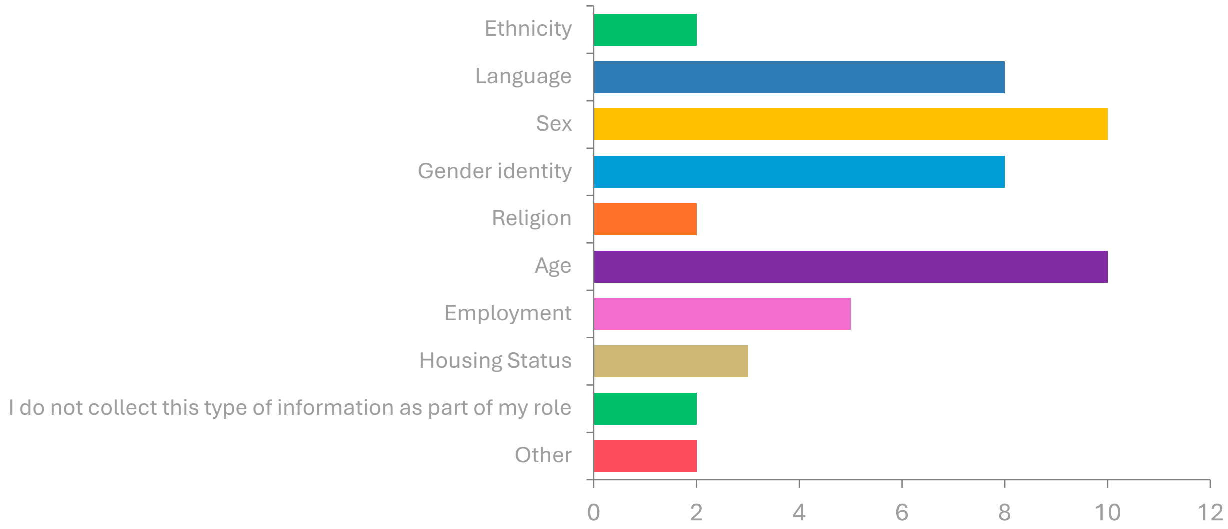

2. What is your opinion on collecting sociodemographic data from patient? Select all that apply.

a. It increases quality of care
b. Not a good idea because of privacy reasons
c. It is too difficult to do
d. It is not relevant for quality of care
e. Other (please specify)

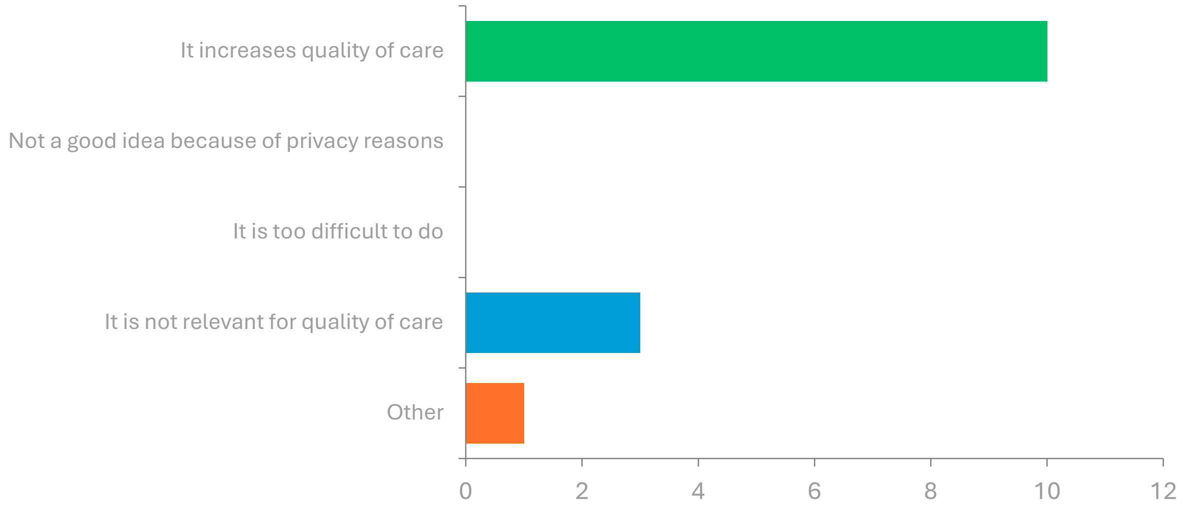

3. What information do you think you need about patients to provide better mental health care? Select all that apply.
   a. Ethnicity
   b. Preferred Language
   c. Sex
   d. Gender Identity
   e. Religion
   f. Age
   g. Employment
   h. Housing Status
   i. Other (please specify)

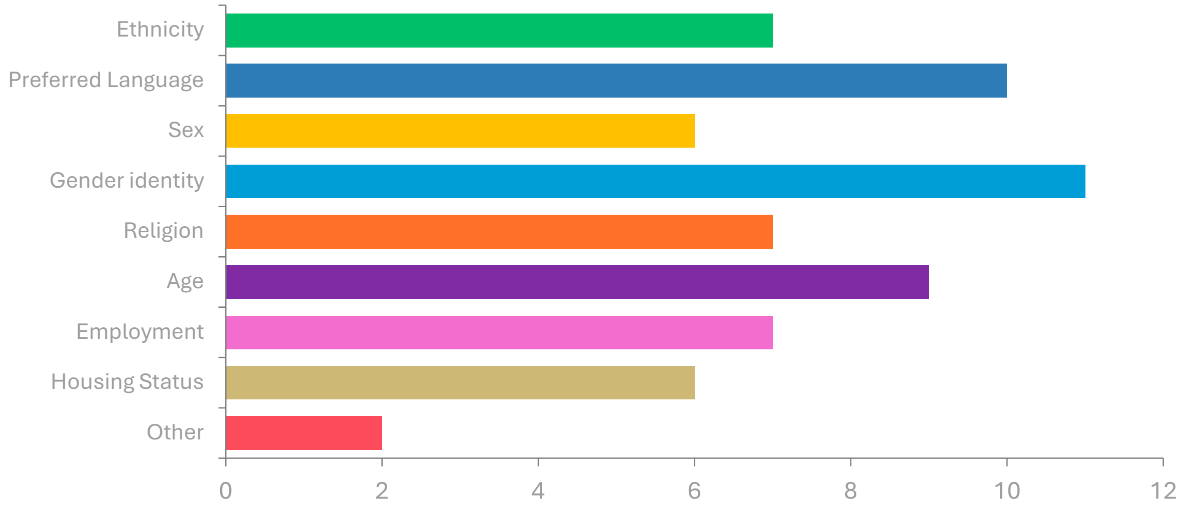

4. How does knowing the ethnic and cultural background of the patient improve their care? Select all that apply.
    a. It increases understanding of patients
    b. It provides access to language interpreters
    c. It creates more timely referrals to specialists and community supports"
    d. It enhances trust between clinician and patient; which increases patient positive engagement with treatment plan
    e. It enhances and improves comprehensive care planning It does NOT matter for patient care
    f. Other (please specify)

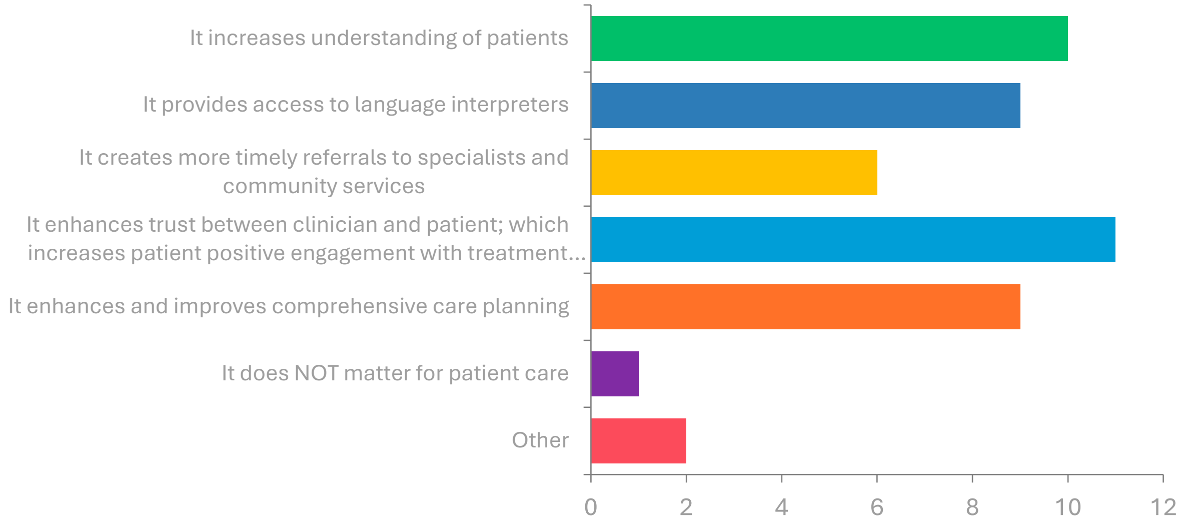

5. What barriers have you experienced when collecting sociodemographic data? Select all that apply.
    a. Patients are reluctant to answer because of privacy concerns
    b. It takes too much time
    c. I feel uncomfortable asking patients for this type of information
    d. I haven't received training in how to ask these types of questions properly

e. Other (please specify)

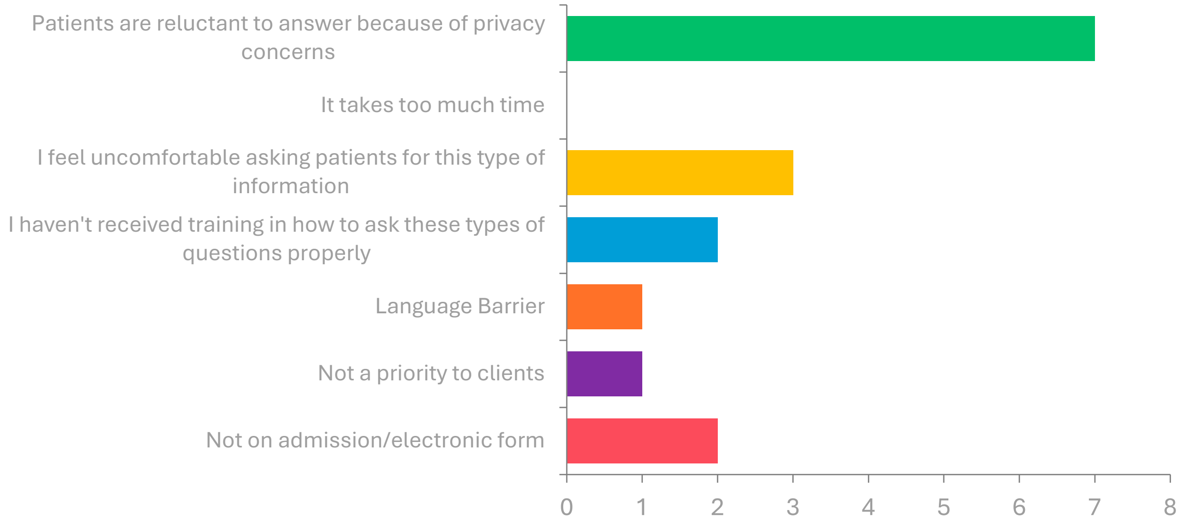

6. What is your role/position at [name of hospital]

7. What are your ancestor's ethnic or cultural origins? Specify as many origins as applicable. Ancestors may have Indigenous origins (e.g., Cree, Métis, Inuit), or origins that refer to different countries (e.g., Scottish, Chinese, Jamaican, Moroccan) or other origins that may not refer to different countries (e.g., Jewish, Acadian, Punjabi).For additional examples, refer to this list of ethnic or cultural origins .

8. What is your current age (in years)?

9. What is your biological sex?
   a. Male
   b. Female

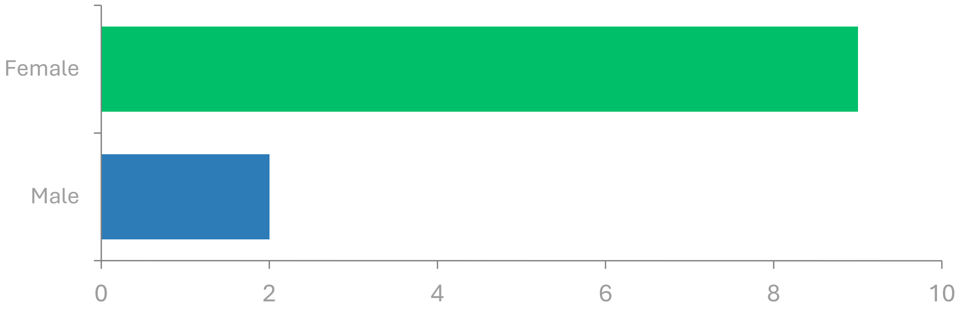

10. Is the gender you identify with the same as your sex registered at birth?
    a. Yes
    b. No
    c. Prefer not to answer
    d. Other: self-describe your gender identity

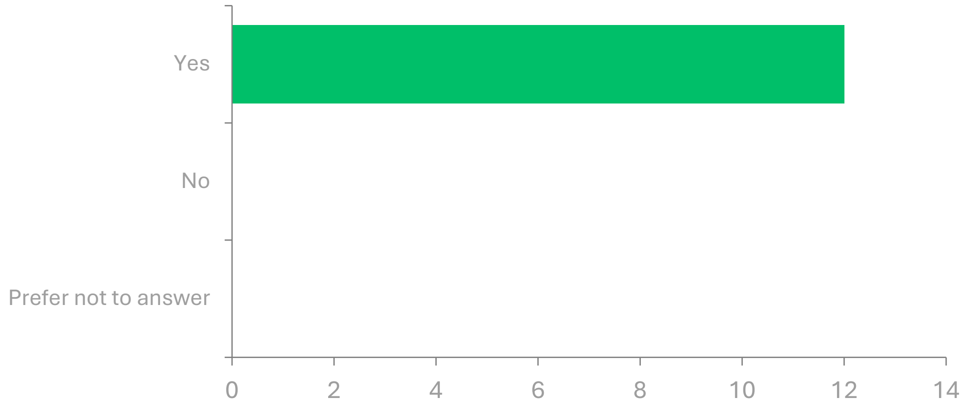

11. What is your sexual orientation?
    a. Asexual
    b. Bisexual
    c. Gay
    d. Heterosexual or straight
    e. Lesbian
    f. Pansexual
    g. Queer
    h. Prefer not to answer
    i. None of the above, please specify

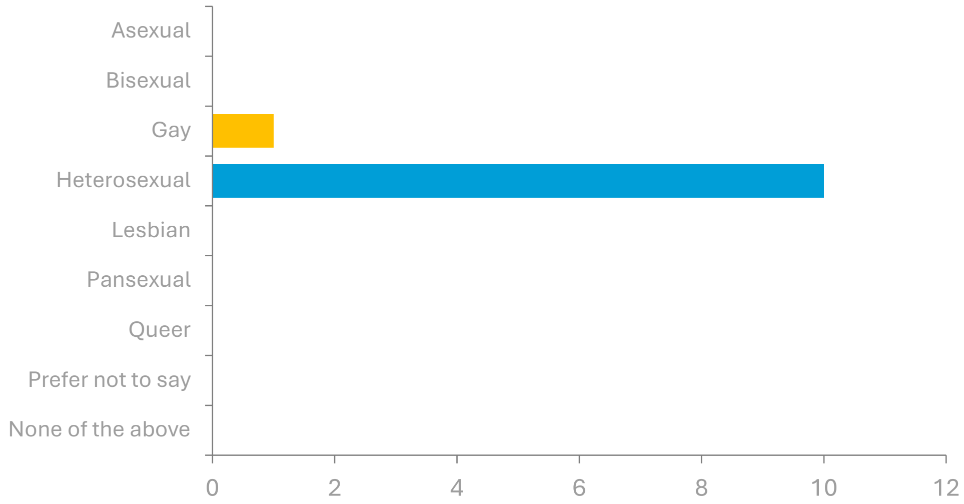

Supplement B

Qualitative Survey Data Summary Codes

B.1 Client Open Responses

Question	Quote	Code
What is your opinion about sharing background information about yourself (e.g. ethnicity, sex, gender, age, etc.) with healthcare providers? (Other please specify)	I didn't explicitly share my ethnicity and still experience labelling snd stereotypes that are imposed on me	discrimination and provider assumptions
	No qualms	no issue
	For statistical purposes and reporting	used for stats and reporting
	Crucial to collect SOGI and/or LGBT2SQ+ self-identification and understand difference between "sex assigned at birth" and "current gender" now with blanks to fill in, not boxes to check , and whether it's even needed in most cases. Best, AFAB ciswoman	gender and sexual identity impacts on care
	Information getting back to my employer VCH. Mental health services	concerns of privacy
	I don't mind answering these questions. I don't think it changes anything.	no issue/perceived impact
	I worry it could improve care for others, but if a health care worker has discriminatory beliefs, it could also impaired someone's care.	can help/hurt depending on provider assumptions
	Dont feel it is relevant and gives an opportunity for someone to be bias	fear of discrimination/feels not useful
	It SHOULD NOT Matter in regards to care I recieve	feels not useful
	I always enter Canadian when they ask Ethnicity	fear of discrimination
	Dunno	Don't know
What information do you think is important for health care providers to have to provide better mental health care for you? (Other please specify)	prior experiences	prior experiences
	not sure	no issue/unknown
	I believe strongly in self-identification that is most meaningful to me, not traditional medical ones	believe in self-identification

	I don't know why these details are necessary to disclose.	needs to be informed
	Family history	family history
	it depends on the reason for seeking care, the goals of care, and what's most important to the patient in terms of their identity and presenting concern	intersecting with mental health/need to be informed
	Information specific to my personal experience affecting my mental health	intersecting with mental health/need to be informed
	Marital status understanding supports in place	social support
	Names of People who support you	social support
	Mental Health issues and assessments	mental health history/need to be informed
	Allergies, Existing heakth conditions	medical history
How does knowing about your background improve your mental health care, if at all? (Other please specify)	It's a critical part of understanding my mental health conditions - not just me as a person, but my actual condition / diagnosis. For example, I live with PTSD caused by trauma that is very specific to my experiences with cultural / ethnic / racial identity. Knowing that information is key for mental health care providers to make accurate clinical assessments of my condition.	Health care providers have a better understanding of me
	you cant put someone in a box and stereo type	shouldn't steortype/categorize people
	Just because they Know, doesn't mean better care WILL be provided though.	consequences/uncertainty of how info will be used
	Ideally this information can lead to more comprehensive, individualized care. It can also lead to stigmatization or refusal of services.	consequences
	Dunno	Don't know
If you have provided background information about yourself to a health care	fear of being judged when I'm totally honest	fear of judgement

provider, what was your experience of sharing the information?<br>(Other please specify)	Due to my ethnicity not being listed on the forms, my answers aren't always the most accurate.	need open response options
	I don't believe in standardized forms over personalized meaning	need open response options
	I was impressed that they asked for gender identity and preferred pronouns	was asked about gender identity
	Did not happen	no issue/unknown
	It seemed to be for funding and eligibility purposes	for funding/eligibility
	Dont recall	no issue/unknown
What makes you feel comfortable sharing your personal information with your healthcare provider?<br>(Other please specify)	I thought it was required so that I can receive care	need to be informed
	I am willing to provide whatever info the provider needs as I assume it will help the quality of my care.	need to be informed
	I believe in an examined, conscious self-identification, respect learned expertise as well as lived/living experience and work in human rights, access and social justice as well as open MHSU peer recovery without "cultural assumptions" and with a priority of public health determinants, anti-colonialism and anti-racism...	lived experience, Health care providers should have a better understanding of me
	No was has ever explained why they are collecting this information.	need to be informed
	I am in an extremely privileged position - I have language privilege (English is my first language), racial privilege (I am white), ethnic privilege (I am a dual citizen of Canada and the UK, and am an atheist), educational privilege (I am trained as a healthcare provider and have a PhD), and socioeconomic privilege (my annual household income is over $450,000)	identifies multiple privileges, makes them feel safe
	How I'm approached	how clients approached

	This is something I would love to experience, but unfortunately never have	never felt comfortable
	Long term trusted relationship w the individual healthcare practitioner	trust is important factor
If you had a bad experience sharing your personal information before with a staff, what made you feel uncomfortable?	not feeling 'heard' or made to my experiences/feelings aren't valid.	not validated/heard
	Staff with similar perceived ethnicity made inaccurate conclusion and assumption about my past based on their own experience	provider assumptions
	Tact/tone while asking, double looking after reading chart	how clients approached
	.	No
	Nothing	No
	N/A	No
	NA	No
	Not getting an explanation	lack of communication/need to be informed
	When staff recognized one half of my ethnic / racial background, but failed to document / mention the other half of my ethnic / racial background in reports. As a result, I felt like a huge part of the context of my mental health history was missing, and it felt awkward to have to try and fill in this missing information with future health care providers who were relying on these incomplete reports.	provider assumptions/incomple te context
	n/a	No
	The way they stereo type and assume and then have you labeled	provider assumptions
	N/A	No
	No	No
	I have felt invisibilized, erased, disempowered, ignored, dismissed, infantilized (ageism, ableism) or assumed to be privileged (able to buy things) with family or a network of	"not validated/heard provider assumptions"

	support. I speak out to correct this, as I'm white, senior, queer, feminist etc. with a lot of middle-class assimilation training and staff goes with their own perceptions and assumptions. At least speaking is a privilege I use responsibility, as it's safer for me and breaks a lot of assumptions and oppressions for others. My self-advocacy usually results in "disqualication" from programs because of "high functioning" but I am multiply disabled on taxable, no case manager, low income cap, no benefits or travel subsidy CPP-Disability (kicked off BC-PWD as I worked all my life). MHSU issue effects and recovery, stroke effects, chronic complex diseases, etc. continue and they are all a part of my life. I also don't take the right kind of pain reliever to gain access re: COVID protection, but am immuno-compromised. Never assume!	
	Their personal biases and judgements based on stereotypes, re: race, ethnicity, gender and age	provider assumptions
	The tone of voice and talking fast couldn't understand	how clients approached
	N/A	No
	N/A	No
	Information is apparent and does not require disclosure ie. Race, SOGI, disability	feels disclosure isnt needed, identity is visible
	N/A	No
	I had only good experiences.	No
	I am always afraid of talking about my partner/emergency contact, and that I will lose my disability income, my relationship because I will become completely financially dependent - I can't work, and end up homeless, sick, and hospitalized, lose everything I have.	fear of discrimination/losing support

	Not knowing why they needed (what felt like irrelevant) personal information.	need to be informed
	Stigma	stigma
	Warm or unfriendly tone	how clients approached
	Yes. I was asked what my "Demographic" is, which confused me. I did not understand what that word meant at the time. During a mental health break. The receptionist continued to repeat the question to me even though I had said I did not understand the question. This happened until the point of me breaking out in tears	how clients approached, not being informed
	I was judged based on my spiritually	provider knowledge and timing of collecting info"
	NA	No
	Feeling judged, dismissed or being denied service made it feel uncomfortable.	judged, ignored, and denied care
	Just the physicians assumptions.	provider assumptions
	For many years The same doctor continued to exhibit signs they did not understand my health issues.	provider didn't understand concerns

B.2 Provider Open Responses

Question	Quote	Code
In your role, what, if any, patient sociodemographic data do you collect? (Other please specify)	Name, Birthday, Next of Kin Contact, Family Doctor, Phone#	confirm basic info/social support
	social connections and social support	social support
What is your opinion on collecting sociodemographic data from patient? (Other please specify)	Understanding how this may be related to their illness and contribute to their care and treatment.	informing care and treatment
What information do you think you need about patients to provide better mental health care? (Other please specify)	Mother's first name	social support
	Social connections and social support.	social suport

How does knowing the ethnic and cultural background of the patient improve their care? (Other please specify)	Unless diseases are specified to that cultural background	related to disease
	Because this plays a major role in understanding client's understanding of what is going on for them so that the most appropriate care and treatment can be implemented.	impacts conceptualization and approach to care
What barriers have you experienced when collecting sociodemographic data? (Other please specify)	LANGUAGE BARRIERS	language
	n/a	none
	none of the above, the client sometimes do not answer as this information is not a priority to them	client priority
	Admission template or admission form does not have all the areas identified in this form thus far.	no admission data
	Telemedicine	no electronic data

Supplement C

Focus Group Questions

Note: *for the group with family members, we will ask them to share about their experience of sociodemographic data being collected about their family member, what their thoughts are about collecting the data, and their ideas for how it can improve practices.*

1. In your experience, has staff at [name of hospital] asked you about your age, gender, ethnicity, and other background information?

2. When during your care was this asked?

3. How was it asked e.g., filled out a questionnaire on admission and/or during intake interview asking about your ethnicity/culture/race?

4. Uncover what people think about collecting socio demographic data (the why)

5. Do you think if care providers knew more about your age, gender, ethnicity, and other background information that it would improve your care? If so, in what ways?

6. From your perspective, does your ethnicity and/or culture influence your mental health or experience with mental health care in any way?

7. Are there things that care providers misunderstand about you if they aren't familiar with your culture/ethnicity? If they knew your culture/ethnicity, in what ways do you think they might be able to support you?

8. Is there any other background information that you think is helpful for your care providers to know in order to provide you with the most appropriate care?

9. Gather ideas about how to improve practices (recommendations)

10. If you could change how your personal information is being collected – what would that be? E.g., when it is being asked or how it is being asked.

11. How could your information be better collected and shared with relevant providers that you work with e.g., when you transition between different care teams?

12. How has COVID-19 impacted mental health?

13. We know that in some cases experiences of racism and discrimination have increased during the pandemic and that this can have a negative impact on mental health.

14. Have you experienced discrimination or racism in the past year? Is so, tell me about that experience? Has it been different from before COVID-19? Why or why not?

15. Would you like to add anything that would be helpful for care providers to understanding how collecting your personal information could improve your mental health?

Supplement D

Data extraction tool for chart review

- [name of database] Extraction
- Patient information
- Demographic review
    - Address – NAF, PA
    - Demographic
    - Marital status
    - Religion
    - Language
    - Disability
    - Employer
    - Next of kin (if social support) did not capture

Results

- Transcriptions
    - Consultations – psychiatrist
    - Discharge summaries for the dates within out time frame
    - Progress Notes – within out time frame
    - Outpatient Transcriptions – if in our time frame
    - Names of referral services recommended were noted

Supplement E

Chart Review Data Table

Start Date	End Date	Age	Gender	Ethnicity-Primary Care	Ethnicity-Community Care	Marital Status	Summary Code
2021-10-16	2021-11-05	33	Male	Caucasian		Single	Addiction referral and previous religious referral
2021-11-11	2021-11-19	22	Female	First Nations		Single	Indigenous navigator, recommended Indigenous clinician, Indigenous community support
2021-08-06	2021-08-10	47	Female	First Nations		M/or Common law	general mental health team, unknown outcomes
2021-09-28	2021-10-05	26	Male	Aboriginal		Single	general mental health team, unknown outcome or culture specific care
2022-02-26	2022-03-08	28	Male	Caucasian		-	several supports e.g. case manager +substance use resource recommended
2021-11-02	2021-11-06	22	Male	Caucasian		Single	general mental health supports and psychiatrist
2021-05-09	2021-06-14	23	Male	Asian		Single	family support, translator for parent, no referrals listed
2021-05-19	2021-05-26	29	Male	Aboriginal		Single	community supports, no culture tailoring
2021-04-02	2021-04-12	38	Male	Caucasian		Single	culture tailored care, follow up for sex and gender care through community health center
2021-08-01	2021-08-06	40	Male	Caucasian		Single	general mental health supports and psychiatrist

2021-11-17	2021-12-31	32	Male	Caucasian		Single	spoke with parent, unclear culture specific care
2022-02-03	2022-02-15	25	Female	Chinese		Single	supports for depression and work only
2021-08-17	2021-09-07	30	Male	Aboriginal		Single	psychosis and substance use support
2021-10-23	2021-11-24	63	Male	Punjabi		Separated	considered language specific support but unclear action/referral taken, spoke with family
2021-05-05	2021-05-25	34	Male	Chinese		Single	spoke to family, no referrals made
2021-07-20	2021-07-23	29	Male	Chinese		Single	psychosis and substance use support
2021-05-21	2021-06-07	27	Female	Aboriginal		Single	community supports, unclear if culture specific
2021-06-17	2021-07-19	40	Male	Caucasian		Single	psychosis and substance use supports, mental health team
2021-04-17	2021-04-26	29	Female	Chinese		Single	mental health team, referral for mental health diagnosis but unclear culture specific care
2021-10-25	2021-12-01	72	Female	Chinese		Widowed	previous mental health team, no clear culture specific care
2021-11-16	2021-11-26	26	Female	Canadian		Single	substance use and suicidality supports discussed, unclear culture specific care
2021-11-14	2022-02-09	27	Male	Canadian	Latin American	Single	mental health supports discussed, unclear culture specific care
2022-03-13	2022-03-17	29	Male	Unknown		Single	community supports, unclear if culturally specific

2021-07-29	2021-08-27	51	Male	Canadian		Single	psychosis supports, general mental health team
2021-10-05	2021-10-11	74	Male	Canadian		Single	followed by clinic
2021-10-24	2021-10-29	36	Female	Other		Divorced	general mental health team, unknown outcome or culture specific care
2021-05-17	2021-06-02	33	Female	-	Caucasian	Single	general mental health team, unknown outcome or culture specific care
2021-04-06	2021-04-12	54	Female	Aboriginal		Widowed	community supports, unclear if culture specific
2021-06-29	2021-07-05	25	Male	Chinese		Single	general mental health supports, unknown outcome or culture specific care
2021-07-12	2021-07-28	51	Male	-	Caucasian	Single	no culturally specific supports, follow up
2022-02-12	2022-02-16	49	Male	Caucasian		Single	ACT and mental health treatment center, frames culturally tailored model of care according to site
2022-02-12	2022-02-16	22	Male	Unknown		Single	spoke to family, psychosis treatment center referral
2021-10-31	2021-11-08	22	Female	-	Caucasian	Single	recommended supports tailored for gender affirming care
2022-02-01	2022-02-05	31	Male	Unknown		Single	substance use support, community referral
2021-10-18	2021-10-23	32	Male	Unknown		Single	community supports, unclear if culture specific
2021-09-10	2021-09-23	50	Male	Canadian		Single	no referrals made; highlighted meds request

2021-01-05	2021-09-07	25	Female	Vietnamese		Single	recommends translator for family, community mental health supports
2021-11-03	2021-12-01	28	Male	Unknown		-	community supports, unclear if culture specific
2022-03-13	2022-03-23	67	Female	Caucasian		Single	community supports, unclear if culture specific
2021-09-23	2021-09-27	33	Female	African		Single	community supports, unclear if culture specific
2022-02-14	2022-03-11	47	Female	Metis		Single	community supports, unclear if culture specific
2021-07-14	2021-07-21	25	Male	Caucasian		Single	community supports, unclear if culture specific
2021-11-05	2021-11-15	33	Male	Caucasian		Single	community supports, unclear if culture specific, substance supports
2021-05-22	2021-06-14	25	Female	-	Caucasian	Single	community supports, unclear if culture specific
2021-04-18	2021-06-30	38	Male	-	Caucasian	Single	community supports, unclear if culture specific
2021-04-18	2021-05-07	29	Male	Aboriginal		Single	psychosis support, unclear if culture specific care
2021-05-10	2021-05-17	24	Female	First Nations		Single	community support and MCFD, unclear if culture specific care
2021-03-20	2021-04-01	58	Male	Chinese		Divorced	no referrals made
2021-09-17	2021-09-24	17	Female	Unknown		Single	community supports, trauma and culture informed care described with session with doctor

2022-01-22	2022-02-10	46	Male	Aboriginal		-	culture specific supports
2021-07-29	2021-07-30	31	Female	Canadian		Married	housing supports
2022-01-11	2022-01-24	23	Male	-	Caucasian	Single	psychosis supports
2021-04-23	2021-04-27	48	Male	Caucasian		Single	general mental health team
2021-11-28	2021-12-01	26	Male	Filipino		Single	general counselling
2022-02-17	2022-02-28	26	Female	French		Single	general mental health team
2022-02-13	2022-02-16	55	Male	German		Married	recommended follow up with mental health team, unclear outcome
2022-02-16	2022-03-01	35	Female	Caucasian		Single	general mental health team
2021-11-04	2021-11-08	27	Female	-	Caucasian	Single	no referrals made
2021-12-01	2021-12-24	49	Male	Caucasian		Divorced	recommended follow up with mental health team, unclear outcome
2021-08-03	2021-08-17	58	Male	Metis		Divorced	general mental health team
2021-05-16	2021-06-02	48	Female	Canadian		Single	general and potentially culturally specific supports but unclear
2021-12-18	2022-01-07	25	Male	Aboriginal		Single	general mental health team

2021-07-26	2021-07-28	55	Female	Aboriginal		s/common law partnership	recommended follow up with mental health team, unclear outcome no other supports offered
2021-09-08	2021-09-17	42	Male	-	Caucasian	lives with male spouse/partner	previous mental health treatment, no referrals made
2021-11-26	2021-12-29	19	Male	African		not specified	recommended follow up for psychosis, unclear outcomes, no other referrals
2021-06-05	2021-06-09	48	Male	Metis NonStatus		Single	no referrals
2021-12-11	2022-01-06	27	Male	African		Single	no referrals made
2022-02-12	2022-02-25	35	Female	Caucasian		Single	has previous case manager, no referrals and unclear outcome
2021-12-13	2021-12-22	35	Female	Aboriginal		Single	previous psychosis supports
2021-04-13	2021-05-19	26	Female	Canadian		Single	psychosis and substance support, community support
2021-06-30	2021-08-09	62	Male	Canadian		Single	general mental health team
2021-10-20	2021-11-24	26	Female	Southeast Asian		married to male partner/separated	previous psychosis supports, no other referrals, unclear outcome
2021-02-22	2021-06-08	29	Male	Canadian		Single	in patient facility, unclear culture supports
2021-04-16	2021-04-17	20	Female	Chinese		Single	community supports and general mental health team

2021-07-11	2021-07-26	70	Male	Caucasian		Married	physical health care, no mental health referrals
2021-07-07	2021-07-12	31	Female	First Nations Status		Married	substance use supports
2022-02-10	2022-03-10	84	Female	Caucasian		Divorced	followed by clinic
2021-05-04	2021-05-12	47	Male	First Nations Status		Single	community supports and general mental health team
2021-08-06	2021-08-12	35	Male	-	Caucasian	Single	community supports and general mental health team
2021-06-19	2021-06-22	21	Male	Chinese		Single	attempted culture specific supports
2021-07-09	2021-07-13	25	Female	Aboriginal		Single	culture specific supports
2022-01-05	2022-01-21	21	Female	Aboriginal		Single	culture specific supports
2021-06-21	2021-08-04	31	Female	Unknown		Single	general mental health team
2021-07-30	2021-07-31	23	Female	Chinese		Single	community supports and general mental health team
